# Exploring *Ocimum basilicum*’s Secondary Metabolites: Inhibition and Molecular Docking against *Rhynchophorus ferrugineus* for Optimal Action

**DOI:** 10.3390/plants13040491

**Published:** 2024-02-08

**Authors:** Hossam Moustafa Darrag, Hesham S. Ghazzawy, Mashail Nasser Alzain, Emadaldeen Hamad Hakami, Hani Taher Almuhanna, Nashi K. Alqahtani

**Affiliations:** 1 Research and Training Station, King Faisal University, Al-Ahsa 31982, Saudi Arabia; ehakmi@kfu.edu.sa (E.H.H.); hmuhana@kfu.edu.sa (H.T.A.); 2 Pesticide Chemistry and Technology Department, Faculty of Agriculture, Alexandria University, Alexandria 21545, Egypt; 3 Date Palm Research Center of Excellence, King Faisal University, Al-Ahsa 31982, Saudi Arabia; hghazzawy@kfu.edu.sa (H.S.G.); nalqahtani@kfu.edu.sa (N.K.A.); 4 Central Laboratory for Date Palm Research and Development, Agriculture Research Center, Giza 12511, Egypt; 5 Department of Biology, College of Sciences, Princess Nourah Bint Abdulrahman University, Riyadh 11451, Saudi Arabia; mnalzain@pnu.edu.sa; 6 Department of Food and Nutrition Sciences, College of Agricultural and Food Sciences, King Faisal University, P.O. Box 400, Al-Ahsa 31982, Saudi Arabia

**Keywords:** green biosynthesis, component molecules, metabolite spectra, abiotic stress, bioactive secondary metabolites

## Abstract

The objective of our work is to create a practical procedure to produce in vitro cell suspensions of *O. basilicum* and to ascertain the factors that encourage enhanced secondary metabolite production. We investigated the impact of these metabolites on *Rhynchophorus ferrugineus*’s adult and larval target enzymes. The explants were cultivated on Murashige and Skoog (MS) media with 0.1 to 1 mg/L plant growth regulators (PGRs) to create calluses. 2,4-Dichlorophenoxyacetic acid (2,4-D), kinetin, 1-naphthylacetic acid (NAA), and indole-3-butryic acid (IBA) at 0.5, 0.5, 0.1, and 1 mg/L, respectively, with 3% sucrose led to the highest biomass accumulation. In cell suspensions, the total phenolic content (TPC) and total flavonoid content (TFC) were 39.68 and 5.49 mg/g DW, respectively, with abiotic *Verticillium dahliae* as an activator. Rosmarinic acid, ursolic acid, nepetoidin A and B, salvigenin, and quercetin-3-O-rutinoside as flavonoids and phenolics were analyzed using UPLC-I TQD MS, with the highest concentrations reached after 40 days. The extract demonstrates insecticidal activity against the fourth-instar larvae of *R. ferrugineus*, with adults at 1197 µg/mL and 12.5 µg/larvae as LC_50_ and LD_50_ values. The extract inhibited acetylcholine esterase (AChE), acid phosphatases (ACPs), alkaline phosphatases (ALPs), and gamma-aminobutyric acid-transaminase (GABA-T) in larval tissue in vitro, with IC_50_ values of 124.2, 149.3, 157.8, and 204.8 µg/mL, and in vivo, with IC_50_ values of 157.2, 179.4, 185.3, and 241.6 µg/mL, after 24 h. Pure compounds identified the activity of the extract, showing the inhibition of AChE, ACPs, ALPs, and GABA-T with IC_50_ values ˂ 200 µg/mL (in vitro). The ABMET examination revealed good oral permeability, and docking tests showed that the compounds bind AChE, ACPs, ALPs, and GABA-T. These findings show that a green bioprocessing method such as an *O. basilicum* cell suspension is a quick and straightforward technique for producing phenolic compounds, and it may be used to develop sustainable bio-insecticides and new green procedures.

## 1. Introduction

*R. ferrugineus* (Oliver) is a harmful phytophagous pest. It was originally discovered along the Gulf Coast toward the tail end of the 20th century, causing major damage to palm trees; subsequently, it began to spread and greatly affected other places [1,2]. Over the last several decades, it has extended to numerous locations, infecting Middle Eastern nations and certain sections of the Mediterranean, North Africa, Central America, and the Caribbean [3,4]. Date palms are susceptible to infection with at least 112 different types of insects and mites [4,5]. Furthermore, *R. ferrugineus* (Oliver) is one of the most serious pests of a broad range of palm species, causing agricultural yield losses in over 40 palm species globally [6,7], including date palms. Because of their ability to spread and pierce palm trees so deeply, the larval stage is one of the most challenging and damaging stages. Larvae feeding on the apical meristem results in the mortality and distortion of palm fronds, which has a disastrous effect on palm plants [8,9,10]. The larvae of the palm weevil create tunnels and make palm trees susceptible to the invasion of various insects and parasites [10,11].

Various chemicals used in pest management have been isolated from plant sources [12], such as fixed, volatile oils; others have potential larvicidal components, e.g., terpenoids, alkaloids, and flavonoids [13]. Biochemicals have been shown to have a significant biocontrol impact against *R. ferrugineus* [14]. Laboratory studies have shown that Invasive Alien is effective as an insecticide against rice weevils [15]. It was demonstrated to have a detrimental effect on protein biosynthesis and enzyme activity, damage DNA [16], and affect enzymatic bioactivity and chitinase efficiency. Scientists have discovered that the latex of *Calotropis gigantea* is an effective pesticide against red palm weevil and an inhibitor of protease and serine protease [16]. Protease inhibitors, as potentially ecologically beneficial agrochemicals, are beneficial for plants and efficient against various biotic threats [11]. Due to their pesticidal action, phenylpropanoids are of particular interest as potential sources for new agents that are harmless to both humans and the environment. The component coumarin also reduces the gene transcription involved in the detoxification process of red palm weevil, indicating that it can serve as a biocontrol agent [17]. Furthermore, picrotoxin can be utilized as a bio-insecticide to control red palm weevil populations [18]. Several possibilities for *R. ferrugineus* population management include geraniol, α-pinene, and 1-octen-3-ol [19], as well as the use of secondary metabolites. Cell suspension extracts of *Thymus vulgaris* demonstrate inhibitory activity on red palm weevil proteinases [11]. Moreover, toxic levels against red palm weevil larvae and pupae are quite high when onion and garlic are combined. Esterase, the expression of glutathione S transferase (GST), and cytochrome P450 genes are all shown to be preferentially suppressed, indicating the mixture’s bioactive activity [20]. *O. basilicum*’s cell suspension extract contains volatile and non-volatile secondary metabolites that demonstrate insecticidal activity against *R. ferruginous*’s adults and larvae [11,21,22]. Spinetoram demonstrates inhibition activity against transaminase and phosphatase enzymes of *R. ferrugineus* in the fat body and hemolymph [23]. *R. ferruginous*’s detoxifying enzymes indicate the potential for the evolution of resistance to *Beauveria bassiana* [24]. Because of their disruptive effects on the enzymatic process, protein, and DNA damage, chitinase from *B. bassiana* and *J. brandegeana* extract has an insecticidal impact against *R. ferrugineus* [2].

The Lamiaceae family, including *O. basilicum*, has been successfully cultivated as a plant species for a very long time in many different countries because of its desirable qualities [23]. Numerous plant genera generate secondary metabolites, including phenols, polyphenols, terpenoids, and flavonoids, e.g., *Ocimum*, and have antibacterial, antioxidant, anti-inflammatory, and bio-insecticidal potential [25,26,27,28,29,30]. Plant secondary metabolites may be produced in a controlled environment in a laboratory using cell and tissue cultures. Due to low efficiency and productivity, plant cells cannot yet reach the bioprocess targets for secondary metabolite production [30,31,32,33]. There has been a comprehensive analysis of potential processes, new directions, and current developments based on plant cells. Tissue cultures and plant cells create secondary metabolites regularly. Still, they cannot generate current productivity and yield levels, so new pathways, plant-cell-based mechanisms, and recent advances are assessed. Genotypes and somatic embryogenesis using various explants, including meristematic cells, have allowed plants to reproduce successfully [8,21]. It is possible to use somatic embryogenesis, which is more efficient, to manufacture secondary metabolites. Cytokinins, auxins, organic additives, amino acids, and baseline salt formulations have been studied as potential means of improving somatic embryogenesis [34,35]. However, few studies have examined how plants synthesize bioactive chemicals in culture. This work extends previous efforts to produce and characterize secondary (volatile) metabolites of the cells of *O. basilicum* and *T. vulgaris* [11,21,22].

Besides agri-industrial food production, basil plant extracts have medicinal and therapeutic purposes [36,37,38]. Numerous studies have revealed that basil extract is a rich antioxidant and health-promoting agent [39,40,41]. The phenolic components in basil mitigate the likelihood of cardiovascular and degenerative ailments by inhibiting oxidative stress and the oxidation of biological macromolecules [42,43,44]. The actions of these components encompass anti-inflammatory, analgesic, antidiabetic effects, antioxidant, hypoglycemic, hepatoprotective, antimicrobial, antihyperlipidemic, antihypertensive, antiulcerative, cardioprotective, immunomodulatory, cytoprotective, cardiostimulant, hypnotic, sedative, anti-nociceptive, chemopreventative, chemomodulatory, anti-convulsant, anti-cancer, anti-parasitic, and larvicidal activities [45,46,47,48]. In agro-industrial food, *Ocimum basilicum* extract has been used as an antimicrobial agent to hinder the growth of bacteria in food, thereby preventing illness resulting from the intake of food contaminated by bacteria such as *Staphylococcus aureus*, *Staphylococcus epidermidis*, *Listeria monocytogenes*, *Pseudomonas putida*, *Pseudomonas aeruginosa*, *Escherichia coli*, *Bacillus subtilis*, and *Shigella* sp. It also demonstrates antifungal activity against aflatoxin-producing *Aspergillus flavus* and yeast species [49,50,51,52]. Due to the recognized and possible harmful effects of chemical food preservatives, natural preservatives are in demand. Food scientists are studying inhibitory substances such as essential oils [53] and plant extracts such as *O. basilicum* extract [54,55] due to the growing need for natural antimicrobials and their importance in antimicrobial packaging due to their perceived lower risk to consumers [56,57].

As indicated earlier, basil plant extracts have several medicinal and therapeutic applications, as well as being used in food production and pest control. Thus, the availability of a commercially viable production technique using tissue culture and cell suspension technology that is ecologically sustainable, efficient, cost-effective, and fast would pave the way for the use of cell suspensions and bioreactors in compound manufacturing. The present study tries to investigate the concept to improve the cell suspension technique for obtaining secondary metabolites from *O. basilicum*. This study also aims to establish a connection between the polyphenolic and flavonoid chemicals found in *O. basilicum* and their potential use as environmentally friendly biopesticides against *R. ferrugineus*. This was determined by assessing their inhibitory effects on AChE, ALP, ACP, and GABA-T enzymes, both in vitro and in vivo. The present study also aims to examine the proliferation of *O. basilicum* cell suspensions supplemented with variant values of PGRs to develop and produce secondary metabolites. LC-MS analysis was used to determine the phenolic content of the components produced from the *O. basilicum* culture. The insecticidal properties of *R. ferrugineus* larvae and adults were examined both in vivo and in vitro to assess their effectiveness as contact insecticides and antifeedants. Additionally, their impact on the red palm weevil’s AChE, ALP, ACP, and GABA-T enzymes was evaluated both in vivo and in vitro. Moreover, we aimed to assess the ADMET property in silico and the process of docking molecules to explain and investigate the effect of *O. basilicum* secondary metabolites with AChE, ALPs, ACPs, and GABA-T. The results are expected to lead to the production of secondary metabolites via the use of a cell suspension as a rapid and simple approach, as well as to the creation of an environmentally benign, natural bio-insecticide for pest management.

## 2. Results

### 2.1. Optimization of Media Amounts and Callus Initiation

The growing conditions for *O. basilicum* using cell and tissue cultures were optimized in the present study. Table 1 displays the findings from an analysis of the callus induction effects of the PGRs KT, IAA, 2,4-D, and IBA. The findings showed that the 2,4-D- and kinetin-supplied (0.1 and 0.5 mg/L, respectively) MS medium had the greatest impact on *O. basilicum* callus development. The proceeding ratio of kinetin to 2,4-D demonstrated 84.7% initiation when surrounded with 1 mg/L of IBA and 83.33% initiation when exposed to an IAA environment of 0.1 mg/L. The callus production frequency was modest in the medium absence of 2,4-D and KT. Most fawn and buff calluses developed in response to the PGR 2,4-D treatment combined with the 0.1 mg/L, and 0.5 mg/L kinetin treatment demonstrated an enhanced propensity to develop into somatic embryos. The medium also experienced an increase in subcultures of fawn and buff calluses with a combination of 2,4-D at varying concentrations with KT for growth and development after 5 or 6 weeks. Following 40 days of treatment using 2,4-D and KT ranging from 0.1 to 0.01 mg/L, the callus transformed into an embryogenic callus.

IBA was used to improve the callus condition. During the callus-forming phase, the IBA concentration was lowered to 0.1 mg/L. According to Table 1, the outcomes of growing cells in media added kinetin and 2,4-D with values 0.1 mg/L. The initiation ratios of kinetin to 2,4-D in growing *O. basilicum* were 60.66% when 1 mg/L IBA was used and 54.66% when 0.1 mg/L IAA was used. In a thirty-day MS medium culture, the hypocotyl segments of the above *O. basilicum* began to expand and create a mass of calluses.

Many concentrations of NAA and kinetin were used to cultivate *O. basilicum*, as shown in Table 2. Callus differentiation was boosted by adding NAA (0.1 or 1 mg/L) and kinetin (0.1 mg/L). Most of the calluses transplanted into this medium failed to form amorphous calluses, showed reduced proliferation, and failed to result in any embryos. In *O. basilicum*, the initiation ratios for kinetin and NAA were 7.33% when using sucrose and 5.66% when using glucose. This medium significantly reduced the amorphousness of the given callus, though not as much as with NAA plus kinetin (1, 0.1 mg/L).

This result demonstrated that, in the case of *O. basilicum*, reducing the NAA concentration while being surrounded by 0.1 mg/L of kinetin improved the outcomes. Table 2 reveals that, although this medium allowed for the maturation and significant embryogenic development of the indicated *O. basilicum*, it did not allow regeneration. It shows that the best NAA and kinetin concentrations were 0.1 and 0.5 mg/L for *O. basilicum* growth. The initialization ratios of kinetin and NAA (0.5 and 0.1 mg/L) were 80.66% with 3% sucrose and 70.66% with 3% glucose. The lowest growth response of *O. basilicum* was seen when the medium included BAP and 2,4-D within 0.5 and 0.1 mg/L, respectively. The initiation ratios were 4.66% with 3% sucrose and 3.33% with 3% glucose. BAP, kinetin, and 2,4-D (0.5 mg/L) resulted in the callus having a medium differentiation ratio. For *O. basilicum*, the above medium initiation ratios were 42.66% with 3% sucrose and 38.66% with 3% glucose. Looking at the additives employed in the modified MS medium revealed that sucrose at a concentration of 3% performed better in its response (Table 2) than glucose. 

When a solid medium was supplemented with PGRs (0.5, 0.5, 0.1, and 1 mg/L of 2,4-D, kinetin, NAA, and IBA, respectively), the modified MS with 3% sucrose and 6.0 g/L of agar was the most efficient medium tested for callus induction (Table 3). Table 3 shows the relative callus weights of *O. basilicum* grown under optimal conditions with and without the activator *V*. *dahliae*. Mean values of callus or cell suspension weights generated at various dates provided clear evidence of *O. basilicum*’s data (Table 3). Following 40 days of growth, the largest possible callus was collected, and the average callus weight increased progressively afterward (Table 3). The callus without *V. dahliae* infection weighed 7.11 g/Petri dish, but the infected callus weighed 10.51 g/Petri dish.

### 2.2. O. basilicum Callus and Cell Suspension Formation

**Figure 1:**
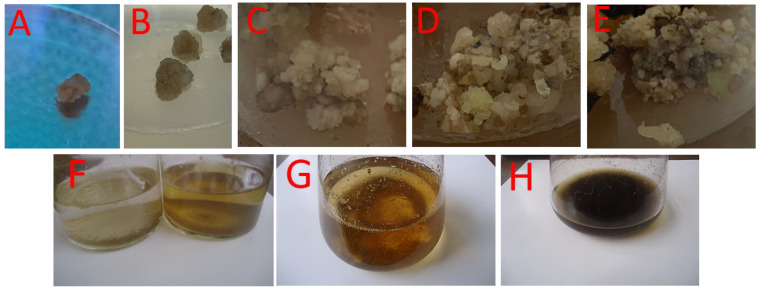
*O. basilicum* callus induction with 2,4-D, NAA, kinetin, and IBA at 0.1, 0.1, 0.5, and 1 mg/L, respectively, and 3% sucrose. (**A**) Callus initiation at day 7 in MS media; (**B**) callus at day 14; (**C**) callus at day 21; (**D**) callus at day 30; (**E**) callus at day 40; (**F**) cell suspension at 1 and 14 days; (**G**) cell suspension at 25 days; (**H**) cell suspension upon completion of induction (40 days); data analysis performed with a one-way analysis of variance (ANOVA).

To initiate the embryonic callus culture, the cell suspension was used. The callus was shown to proliferate and be ready for transfer to the media for cell suspension at between five and six weeks of age. In contrast to the solid medium employed for this reason, the LS liquid media consistently generated more somatic embryos. Over the course of 5–6 weeks, embryonic calluses were grown in a cell suspension culture. They multiplied faster and started more somatic embryos than could have been achieved on a solid medium. Differences in *O. basilicum* cell suspension densities between infected and uninfected *V. dahliae*-grown cultures were presented. After 40 days, the uninfected and infected cell suspensions reached their maximum weights (6.63 g/200 mL of medium). Table S1 shows that, across the board, callus weights were higher than those measured directly from cell suspensions. *O. basilicum’s* cell suspension gained significant weight after being infected with *V. dahliae*. When grown under conditions free of *O. basilicum* infection, the callus weight peaked at 8.94 g after 40 days. Weight values rose further with callus age and *V. dahliae* infection. *O. basilicum* had 11.29 g of infected calluses (Table S1). However, the calluses underwent an oxidative procedure that rapidly resulted in brown coloration, revealing that just a fraction of the somatic embryos produced developed when given sufficient macronutrients (Figure 1). Initiation was characterized by a brownish hue in the liquid media and the calluses, which became darker with time. These findings demonstrate that time is essential for both the initiation technique and phenolic compound production in the environment.

### 2.3. Extract of O. basilicum: A Phytochemical Study

By the conclusion of the 40-day test period, both the infected *O. basilicum* calluses’ phenolic content and the cell suspension increased, peaking at 21.75 and 39.68 mg/g dry weight, respectively, after infection with *V. dahlia* (Table 4). No infection was detected in the callus or cell suspension results, which yielded 12.48 and 19.31 mg/g of dry weight, respectively. *O. basilicum* infected and uninfected with *V. dahliae* strongly influenced the total phenolic content (*p* ≤ 0.0005) (Table 4). A greater number of total flavonoids (5.49 mg /g DW) was detected in the *V. dahlia*-infected cell suspension, as shown in Table 4. The result was 1.47 mg/g DW lower in the callus that was not infected.

### 2.4. Application of UPLC–I Class Combined with Xevo TQD MS for Flavonoids and Phenolics Components Analysis in Extract of Cell Suspension O. basilicum

To quantify the flavonoids and phenolics in the extract of cell suspension, their molecular weight, retention time, and fragmented character were characterized with data from the literature and a software database providing confirmation (Table 5). Using the mass spectrometer’s negative scan mode, the discovered chemicals were identified during the scan, which lasted 45 min.

Rosmarinic acid, a polyphenol (15.94 mg/g DW), had the highest concentration in the extracts; it was identified using *m*/*z* 117, 135, 161, 179, and 197 with a retention period of 11.58 min (rosmarinic acid). An extract containing both nepetoidin A and B was shown, with respective concentrations of 7.35 and 6.21 µmol g^−1^ per cell. Nepetoidin A and B, each with an *m*/*z* of 314.29, were retained for 25.51 and 25.64 min (M-H). For nepetoidin A, the *m*/*z* was 133, 161, 313, and 335, which was similar to that of nepetoidin B, for which the *m*/*z* value ranged from 133 to 161, 269 to 313, and 335 to 335. Ursolic acid, a polyphenol, was found at 5.12 µmol g^−1^ per cell in the extract. The resulting ions had masses of 455, 456, 523, 524, and 591, and the retention time at an *m*/*z* value of 456.7 (M-H) was 26.03 min. Salvigenin was found at 2.86 µmol g^−1^ per cell in the extract. The resulting ions had masses of 116.9, 205, 215, 277, and 311, and the retention time at a *m*/*z* value of 327.215 (M-H) was 18.26 min. Quercetin-3-O-rutinoside was found at 2.67 µmol g^−1^ per cell in the extract. The resulting ions had masses of 465, 449, and 303, and the retention time at an *m*/*z* value of 611.16 (M-H) was 11.50 min.

The cell suspension extract contained rosmarinic acid glucosides A and B at concentrations of 1.97 and 1.75 µmol g^−1^ per cell, respectively, with an *m*/*z* of 521.12 (M-H). Ions with the following mass numbers were observed for rosmarinic acid glucoside A: 135, 161, 179, 197, and 359; for rosmarinic acid glucoside B, ions with the following mass numbers were obtained: 135, 161, 179, 197, 323, and 359, respectively, with 21.35 and 25.12 min as retention times. The extract contained 1.31 µmol g^−1^ per cell of nepetoidin glucoside, with a retention period of 27.93 min an *m*/*z* of 475.12 (M-H). Ions with the given mass numbers were detected with an *m*/*z* of 151, 161, 313, 323, and 475. Isocitric and chicoric acid were abundant in extract containing 1.62 and 1.32 µmol g^−1^ per cell, with chicoric acid detectable at *m*/*z* values of 473, 311 (293), and 179 (149) with a 11.16 min retention period. Isocitric acid was detected according to the obtained retention time of 2.53 min and a molecular mass of 191.01 (M-H), yielding ionic masses of 111, 129, and 173. Several substances were found in the extracts, including (with the obtained *m*/*z* (M-H)) apigenin 7-O-glucoside (432.4), naringenin 7-0-glucoside (434.4), cyanidin 3-O-rutinoside (595.17), and cyanidin 3,3′-diglucoside (611.16). The relevant compounds were identified as salvianolic acid with *m*/*z* values in extracts of salvianolic acid F (313.07), salvianolic acid B (717.15), salvianolic acid A (394.11), salvianolic acid E (717.15), salvianolic acid H/I (537.10), and salvianolic acid K (555.11). The *m*/*z* values for the other acids discovered were 537.10, 359.70, 325.06, 311.04, 179.03, and 149.0076 for lithospermic acid, caffeic acid derivative (3TMS), fertaric acid, caftaric acid, caffeic acid, and tartaric acid, respectively [21,58,59,60,61,62,63,64,65,66,67,68,69,70,71].

**Figure 2:**
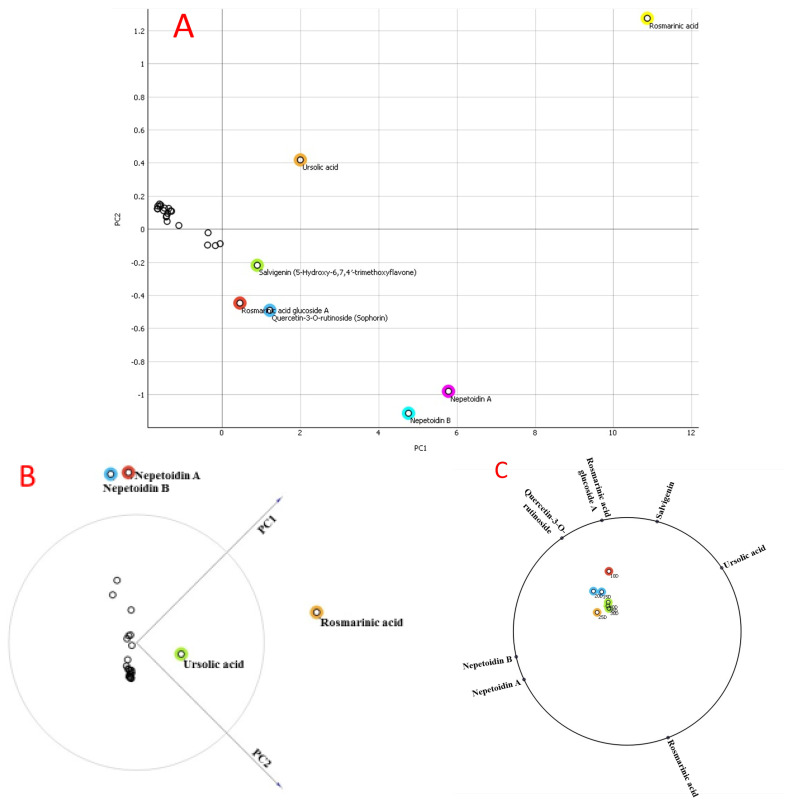
The metabolite spectra of 40-day-old cell suspension extracts analyzed using principal component analysis (PCA) to explore the initiation of the metabolic process; loading plot (**A**); score plots (**B**,**C**).

To verify how metabolites play a part in the clustering algorithm and how there were discrepancies across samples, principal component analysis (PCA) was used. No difference could be seen in Figure 2 between the various *O. basilicum* extracts. PC1 explained 96.70% of the variation, whereas PC2 explained just 3.30%. *O. basilicum* extracts were rich in acidic chemicals, including chicoric acid and isocitric acid, as well as the secondary metabolites salvigenin, quercetin-3-O-rutinoside, nepetoidin A and B, ursolic acid, rosmarinic acid, nepetoidin glucoside, and rosmarinic acid glucoside A and B (Figure 2). The importance of the correlations between specimens and the roles played by the metabolites was assessed using PCA. A large variation between examples showed a clear difference (Figure 2).

**Figure 3:**
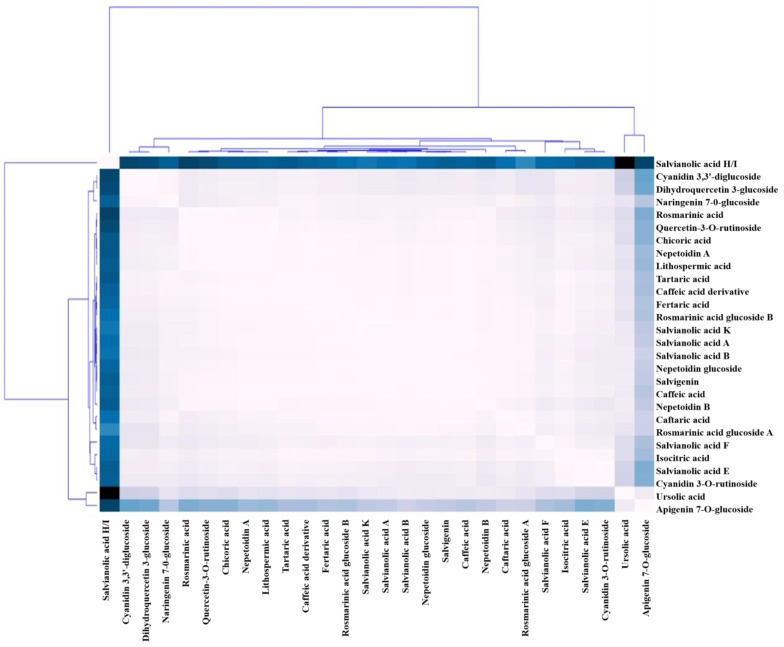
Compound data from *O. basilicum* cell suspension extracts were analyzed using hierarchical clustering analysis (HCA) and absolute Pearson correlation heatmap distance (APCHD) (40 days) with the intensity of the lightest or darkest blue. Pearson correlation coefficients for pairs of chemicals are shown graphically as “squares” with each pair of molecules. The whole graph displays the overall relationship between all pairs of chemicals.

According to the loading plot (during the previous 15 days), 40 days of fasting in a cell suspension led to contents (µmol g^−1^ per cell) being dominated by the metabolites rosmarinic acid (15.94), nepetoidin A (7.35), nepetoidin B (6.21), ursolic acid (5.12), salvigenin (2.8), and quercetin-3-O-rutinoside (2.67), followed by rosmarinic acid glucoside A, isocitric acid, rosmarinic acid glucoside B, chicoric acid, and nepetoidin glucoside with contents of 1.97, 1.62, 1.75, 1.32, and 1.31 µmol g^−1^ per cell, respectively. After 40 days of cell suspension, the maximum amounts of rosmarinic acid, nepetoidin A and B, ursolic acid, salvagingin, and quercetin-3-O-rutinoside were found in the samples, according to this research. The average linkage and Pearson’s correlation were used in a hierarchical clustering analysis (HCA) to look for correlations between the concentrations of the 28 metabolites, and the results indicated values for polyphenolic acids, acid derivatives (18 substances), and flavonoid compounds, among others (10 compounds). The proximity of metabolites in HCA indicated a strong relationship between them (Figure 3). Figure 3 further demonstrates that the growth of *O. basilicum* components was gradual throughout the first 25 days in the cell solution. On the last ten days (after 40 days), they rose faster (Figure 3).

### 2.5. Effectiveness against Adult and Larval R. ferrugineus Insect Extract and Tested Compounds from O. basilicum

For *R. ferrugineus* adults, as shown in Table 6, the extract presented an LC_50_ value of 1197 g/mL and 1086–1291 at a confidence limit of 95%. Chicoric acid, ursolic acid, salvigenin, quercetin-3-O-rutinoside, salvianolic acid B, rosmarinyl glucoside, salvianolic acid A, and nepetoidin B demonstrated the highest insecticidal efficacy according to an LC_50_ of 1097, 1121, 1143, 1168, 1195, 1228, 1261, and 1273 µg/mL, with 1016–1175, 1015–1213, 1017–1226, 1064–1231, 1121–1278, 1126–1294, 1197–1315, and 1227–1314 values at 95% confidence limits, respectively. The LC_50_ values for moderately low activity in adults were 1427, 1492, 1527, and 1795 g/mL for rosmarinic acid, caftaric acid, fertaric acid, and isocitric acid, with 1318–1489, 1382–1546, 1468–1617, and 1714–1879 at 95% confidence limits, respectively.

*O. basilicum* extracts applied topically had an LD_50_ (in micrograms per larva) of 12.5. The maximum insecticidal action was observed for chicoric acid (9.45 µg/larva), salvigenin (10.8 µg/larva), salvianolic acid B (11.1 µg/larva), salvianolic acid A (11.3 µg/larva), nepetoidin B (11.5 µg/larva), and rosmarinic acid (11.8 µg/larva), in that order. The larvae showed moderate sensitivity to ursolic acid (14.9 µg/larva), quercetin-3-O-rutinoside (16.2 µg/larva), and rosmarinyl glucoside (17.2 µg/larva). Lastly, the LD_50_ values for caftaric acid, fertaric acid, and isocitric acid were 18.79, 19.46, and 22.7 µg/larva, respectively, indicating low action.

### 2.6. Assessment of Extract and Compounds O. basilicum (Basil) and Their Effects on AChE, ALPs, ACPs, and GABA-T (In Vitro)

**Figure 4:**
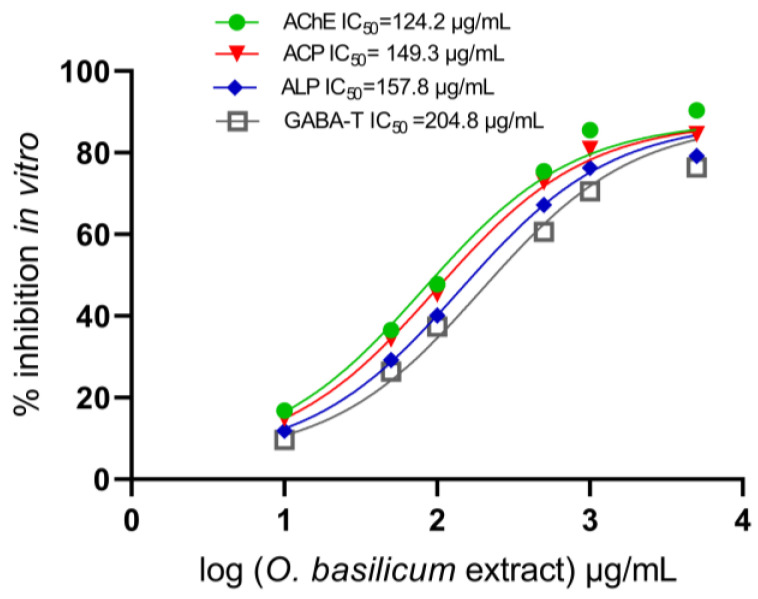
IC_50_ values for AChE, ALPs, ACPs, and GABA-T (in vitro) when compared with the *O. basilicum* extract with a one-way ANOVA.

Methanol extracts were tested alongside major identified compounds, including rosmarinic acid, ursolic acid, nepetoidin B, quercetin-3-O-rutinoside, salvigenin, rosmarinyl glucoside, chicoric acid, isocitric, caftaric, fertaric, salvianolic acid A, and salvianolic acid B, to explore whether they could act as anticholinesterase inhibitors. The data showed that IC_50_ values were affected by successive dosages of *O. basilicum* extract compared to those of untreated acetylcholinesterase from the larvae. The IC_50_ value was elevated with an increased dosage, as shown in Figure 4. Extracts of *O. basilicum* had an IC_50_ with AChE of 124.2 g/mL and optical density (OD) at 0.59 per mg of protein. The AChE-specific activity, measured as the optical density (OD) per milligram of protein (mg), was similar in the complete homogenate preparations. Extracts of *O. basilicum* had a substantial impact on all IC_50_ values. Midgut homogenate preparations showed that *O. basilicum* extract inhibited AChE, ACPs, and ALPs with much higher IC_50_ values than GABA-T. Figure 4 displays the calculation of the IC_50_ of the extract, illustrating the wide range of inhibition in homogenates. The ACP and ALP activity was 0.61 and 0.59 OD.mg protein^−1^ min^−1^, respectively, during the instar’s preparation of the midgut. *O. basilicum* extract was also effective, with IC_50_ values of 149.3 and 157.8 µg/mL. Figure 4 displays the IC_50_ values for GABA-T, showing that the extract inhibits GABA-T to a reduced extent. The IC_50_ value was 204.8 µg/mL with a 0.74 OD/mg protein min.

Table 7 shows the effects on AChE, ACP, ALP, and GABA-T activity in vitro obtained from fourth-instar larvae. All the substances studied had significantly different IC_50_ values. An increased level of activity and effectiveness against AChE was shown for the tested compounds of rosmarinyl glucoside, salvianolic acid A, quercetin-3-O-rutinoside, chicoric acid, salvianolic acid B, rosmarinic acid, fertaric acid, salvigenin, nepetoidin B, caftaric acid, and ursolic acid, with IC_50_ values of 89.4, 93.6, 98.4, 107.5, 116.7, 119.2, 123.6, 129.7, 147.2, 156.4, and 169.5 µg/mL, respectively. Isocitric acid was only moderately effective against AChE (IC_50_ = 197.3 µg/mL).

The IC_50_ values for inhibiting ACP activity ranged from 98.2 to 112.2 g/mL for salvianolic acid B, chicoric acid, quercetin-3-O-rutinoside, salvianolic acid A, rosmarinyl glucoside, fertaric acid, rosmarinic acid, caftaric acid, salvigenin, nepetoidin B, ursolic acid, and isocitric acid (98.2, 101.7, 104.8, 112.2, 123.6, 133.5, 139.7, 146.8, 151.2, 154.2, 167.8, and 192.4 µg/mL, respectively).

ALP inhibited quercetin-3-O-rutinoside, salvianolic acid B, salvianolic acid A, rosmarinyl glucoside, salvigenin, chicoric acid, fertaric acid, ursolic acid, rosmarinic acid, caftaric acid, nepetoidin B, and isocitric acid specifically and strongly (IC_50_ ˂ 200 µg/mL). The IC_50_ values of the most effective substances were 102.3, 104.6, 115.8, 119.7, 122.8, 129.7, 137.2, 141.3, 149.7, 157.6, 166.3, and 194.7 g/mL, respectively (Table 7).

Salvigenin had an IC_50_ value of 147.3 µg/mL for inhibiting GABA-T activity. Additionally, the IC_50_ values for salvianolic acid B, salvianolic acid A, and rosmarinyl glucoside toward GABA-T were 197.2, 213.8, and 248.7 µg/ mL, respectively. The IC_50_ levels for GABA-T are shown in Table 7, indicating that some substances had a lesser inhibitory effect with an IC_50_ of more than 300 µg/mL. Moreover, GABA-T was inhibited via rosmarinic acid, chicoric acid, quercetin-3-O-rutinoside, isocitric acid, ursolic acid, nepetoidin B, caftaric acid, and fertaric acid components in accordance with values of 325.4, 336.2, 347.4, 358.9, 369.1, 374.2, 386.1, and 412.3 µg/mL, respectively.

### 2.7. The Impact of Basil (O. basilicum) Extract on the Activity of AChE, ALPs, ACPs, and GABA-T in Fourth-Instar R. Ferrugineus Insects In Vivo

**Figure 5:**
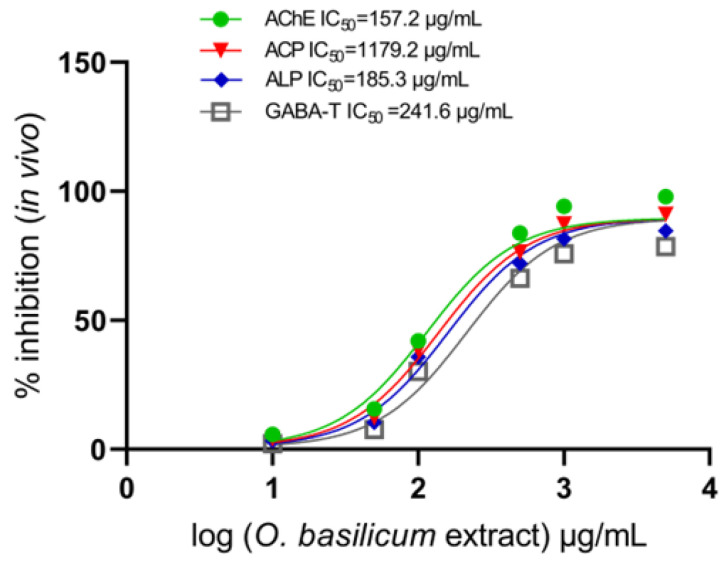
IC_50_ values for AChE, ALPs, ACPs, and GABA-T (in vivo) when compared with the *O. basilicum* extract with one-way ANOVA.

At increasing quantities of *O. basilicum* extract, the IC_50_ value in *R. ferrugineus* larvae in the fourth instar showed a progressive rise in activity. According to the IC_50_ value, *O. basilicum* extracts exerted influentially large effects (Figure 5; Table 8). The AChE, ACP, ALP, and GABA-T values from the fourth preparation showed inhibition via the *O. basilicum* extract, and they were all greater than the in vitro values. Using IC_50_ values of 157.2, 179.4, 185.3, and 241.6 µg/mL, the extract of *O. basilicum* reacted actively against AChE, ACPs, ALPs, and GABA-T. The IC_50_ values were 178.2, 211.6, 229.7, 235.1, 258.4, and 281.7 µg/mL for salvianolic acid A, chicoric acid, salvianolic acid B, rosmarinic acid, salvigenin, and nepetoidin B, respectively, suggesting that they demonstrated activity against AChE. The activity against AChE revealed that substances had the lowest inhibition when their IC_50_ values were 312.7, 347.6, 384.7, 429.2, 457.8, and 494.2 µg/mL for rosmarinyl glucoside, quercetin-3-O-rutinoside, fertaric acid, caftaric acid, ursolic acid, and isocitric acid, respectively.

The compounds with the greatest inhibition (IC_50_ ˂ 330 µg/mL) against ACPs were salvianolic acid B, chicoric acid, salvianolic acid A, rosmarinic acid, salvigenin, and nepetoidin B. Their respective IC_50_ values were 182.3, 204.8, 226.3, 258.7, 294.2, and 294.3 µg/mL. The lowest IC_50_ values against ACPs were 298.4, 318.8, 349.4, 376.9, 392.4, and 415.6 µg/mL, respectively, for quercetin-3-O-rutinoside, rosmarinyl glucoside, fertaric acid, caftaric acid, ursolic acid, and isocitric acid. With corresponding IC_50_ values of 194.6, 213.8, 228.7, 237.2, 256.7, 297.2, and 307.9 µg/mL, salvianolic acid B, salvianolic acid A, salvigenin, chicoric acid, rosmarinic acid, caftaric acid, and nepetoidin B demonstrated more significant activity against ALPs. The IC_50_ values (µg/mL) for Quercetin-3-O-rutinoside (348.2), rosmarinyl glucoside (387.4), fertaric acid (436.8), ursolic acid (476.9), and isocitric acid (448.3) against ALPs were all low, and they all had a lower impact. To inhibit GABA-T activity, salvigenin had an IC_50_ of 234.6 µg/mL; the lowered activity was shown in the IC_50_ values of 324.8 µg/mL for salvianolic acid B and 376.4 µg/mL for salvianolic acid A. The weakest inhibitory effects were shown with rosmarinic acid, chicoric acid, nepetoidin B, rosmarinyl glucoside, quercetin-3-O-rutinoside, isocitric acid, ursolic acid, caftaric acid, and fertaric acid, whose IC_50_ values were more than 500 µg/mL (523.9, 576.8, 615.4, 663.8, 707.4, 749.2, 792.1, 845.3, and 914.2 µg/mL, respectively).

### 2.8. Compound–Enzyme Docking

#### 2.8.1. Acetylcholine Esterase (AChE) Docking

**Figure 6:**
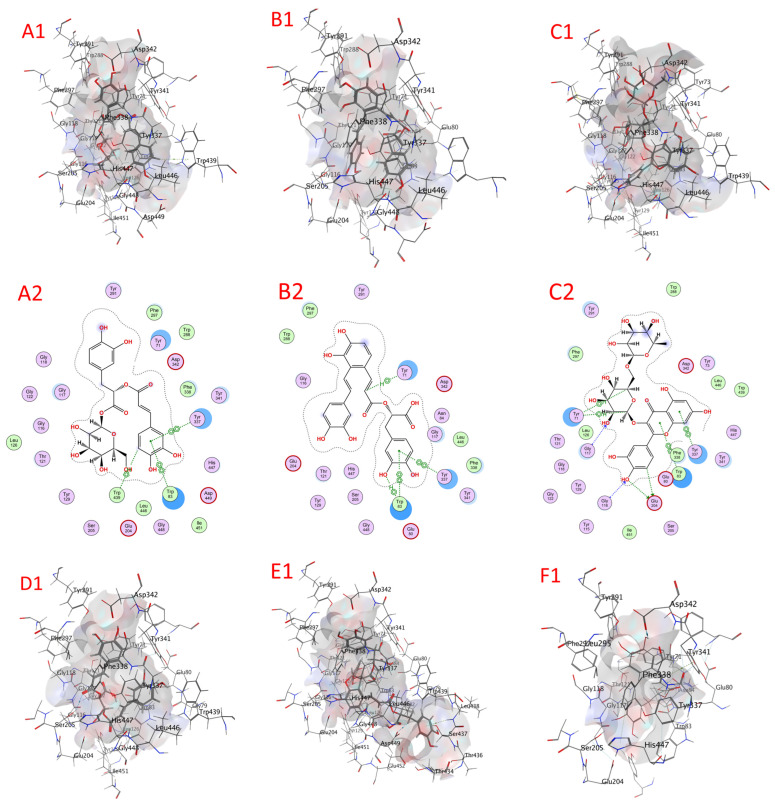

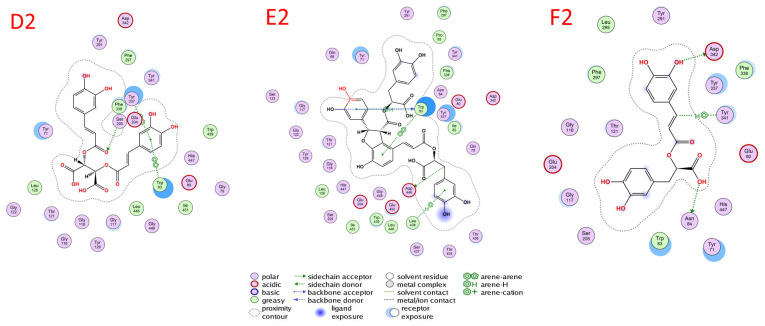
A docking image of the binding sites of acetylcholinesterase AChE (1QON) with rosmarinic acid glucoside (**A1**,**A2**), salvianolic acid A (**B1**,**B2**), quercetin-3-O-rutinoside (**C1**,**C2**), chicoric acid (**D1**,**D2**), salvianolic acid B (**E1**,**E2**), and rosmarinic acid (**F1**,**F2**); complex three-dimensional (stereoview) structures (**A1**–**F1**) and two-dimensional (**A2**–**F2**) interaction maps.

The compounds’ docking scores with AChE are shown in Table 9 and Table S2, as well as Figure 6 and Figure S2 (1QON). The examined compounds showed low docking energy for the target enzyme (AChE), with values from −4.9998 for isocitric acid to −9.4433 for rosmarinic acid glucoside kcal/mol, as determined via docking analysis (Table 7). Compounds with docking energies of approximately −9.4433 kcal/mol had greater binding affinities than those with the lower approximate value of −7.3476 kcal/mol, including rosmarinic acid glucoside, salvianolic acid A, quercetin-3-O-rutinoside, chicoric acid, salvianolic acid B, and rosmarinic acid, with values of −9.4433, −9.2815, −8.6174, −8.3067, 7.5902, and −7.3476 kcal/mol, respectively. Accordingly, fertaric acid, salvigenin, nepetoidin B, caftaric acid, nepetoidin A, and ursolic acid seemed to have the lowest sensitivity for AChE with values of −7.1210, −7.0150, −6.3910, −6.3860, −6.2567, and −6.2136 kcal/mol. Finally, isocitric acid had the lowest docking energy (−4.9998 kcal/mol) (Table 7).

#### 2.8.2. Acid Phosphatase (ACP) Docking

**Figure 7:**
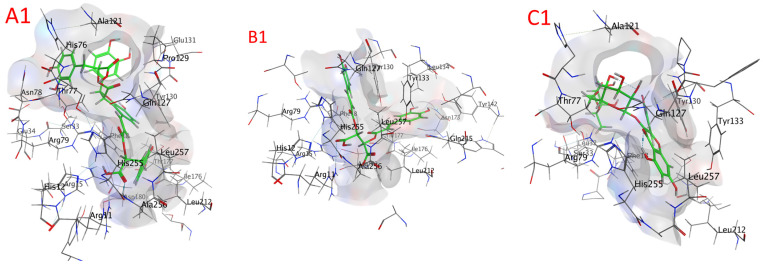

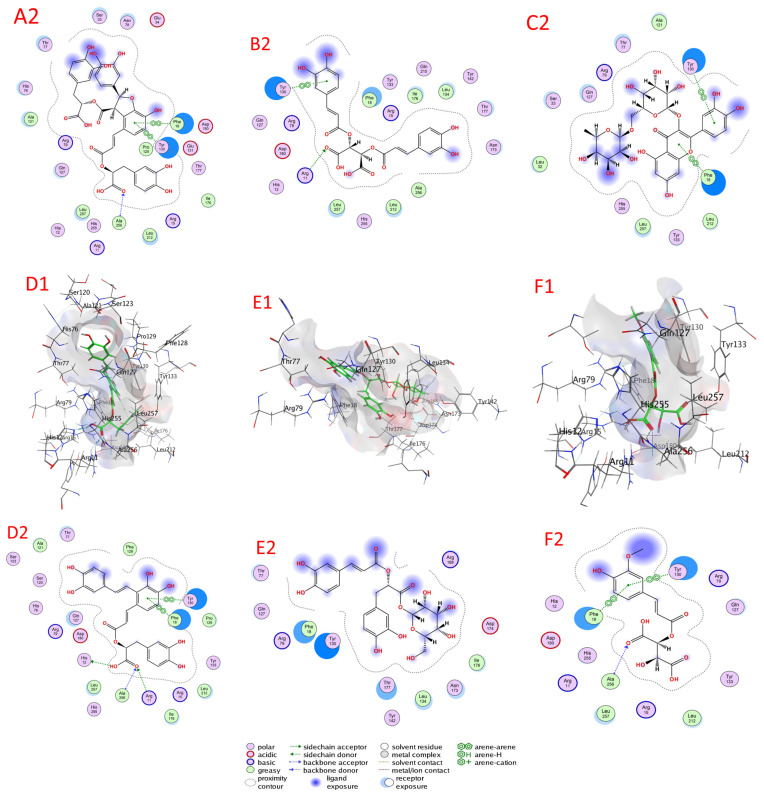
A docking image of the binding sites of acid phosphatase (ACP) (3IT3) with salvianolic acid B (**A1**,**A2**), chicoric acid (**B1**,**B2**), quercetin-3-O-rutinoside (**C1**,**C2**), salvianolic acid A (**D1**,**D2**), rosmarinic acid glucoside (**E1**,**E2**), and fertaric acid (**F1**,**F2**); complex three-dimensional (stereoview) structures (**A1**–**F1**) and two-dimensional (**A2**–**F2**) interaction maps.

Compounds’ docking scores with ACP are shown in Table 9 and Table S3, as well as Figure 7 and Figure S3 (3IT3). The examined compounds showed low docking energy for the target enzyme (ACP), with values ranging from −5.4562 (isocitric acid) to −9.5805 (salvianolic acid B) kcal/mol, as determined via docking analysis (Table 7). Compounds with docking energies of approximately −9.5805 kcal/mol had greater binding affinities than those with a lower value of approximately −7.3173 kcal/mol, including salvianolic acid B, chicoric acid, quercetin-3-O-rutinoside, salvianolic acid A, rosmarinic acid glucoside, and fertaric acid, with values of −9.5805, −9.4951, −8.8978, 8.3457, 7.6360, and −7.3173 kcal/mol, respectively. Hence, rosmarinic acid, caftaric acid, salvigenin, nepetoidin B, nepetoidin A, ursolic acid, and isocitric acid seemed to have the lowest sensitivity to the ACP enzyme with values of −7.0730, −6.9919, −6.8416, −6.8358, −6.6863, and −6.2674 kcal/mol. Isocitric acid had the lowest docking energy (−5.4562 kcal/mol) (Table 7).

#### 2.8.3. Alkaline Phosphatase (ALP) Docking

**Figure 8:**
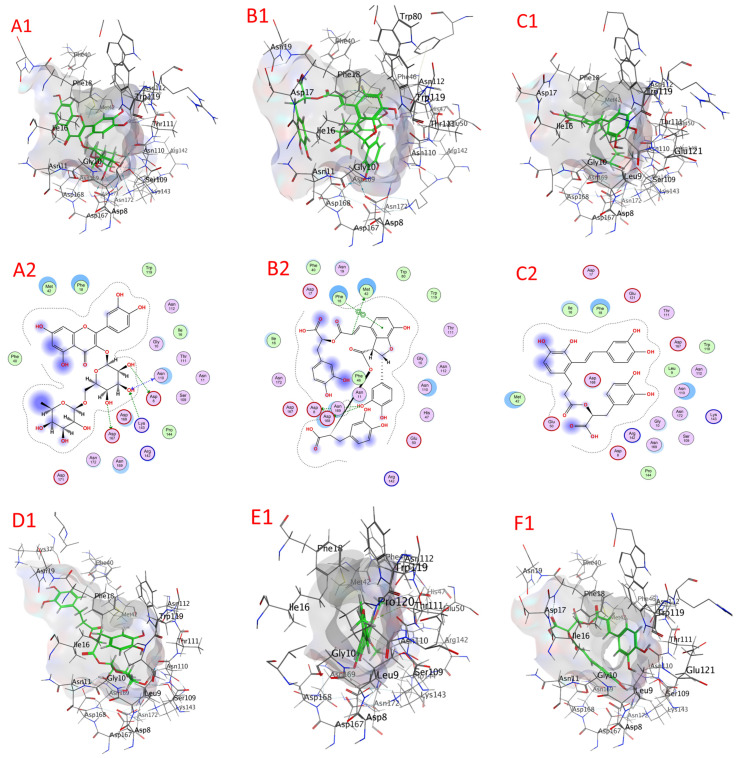

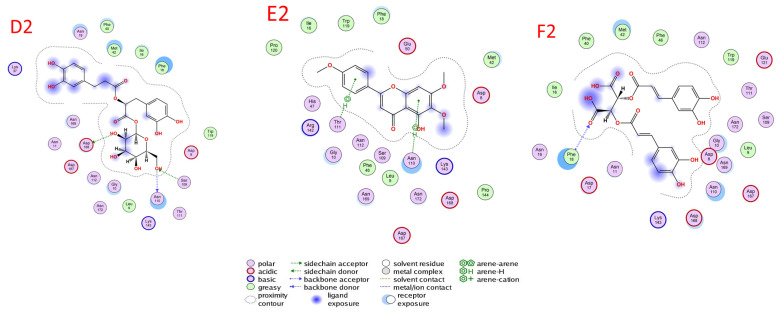
A docking image of the binding sites of alkaline phosphatase (ALP) (2B0C) with quercetin-3-O-rutinoside (**A1**,**A2**), salvianolic acid B (**B1**,**B2**), salvianolic acid A (**C1**,**C2**), rosmarinic acid glucoside (**D1**,**D2**), salvigenin (**E1**,**E2**), and chicoric acid (**F1**,**F2**); complex three-dimensional (stereoview) structures (**A1**–**F1**) and two-dimensional (**A2**–**F2**) interaction maps.

Compounds’ docking scores with ALP are shown in Table 9 and Table S4, as well as Figure 8 and Figure S4 (2B0C). The examined compounds showed low docking energy for the target enzyme (ALP), with values ranging from −5.2278 (isocitric acid) to −8.9556 (quercetin-3-O-rutinoside) kcal/mol, as illustrated via the docking analysis (Table 7). Compounds with docking energies of approximately −8.9556 kcal/mol were more likely to bind compared to those with a lower value of approximately −7.5352 kcal/mol, including quercetin-3-O-rutinoside, salvianolic acid B, salvianolic acid A, rosmarinic acid glucoside, salvigenin, and chicoric acid, with values of −8.9556, −8.8400, −8.0411, −7.7995, −7.6869, and −7.5352 kcal/mol, respectively. Thus, fertaric acid, ursolic acid, rosmarinic acid, caftaric acid, nepetoidin B, nepetoidin A, and isocitric acid seemed to have the lowest sensitivity for the ALP enzyme with values of −7.1331, −7.0948, −6.9925, −6.5749, −6.2554, −5.7638, and −5.2278 kcal/mol (Table 7).

#### 2.8.4. Gamma-Aminobutyric Acid–Transaminase (GABA-T) Docking

**Figure 9:**
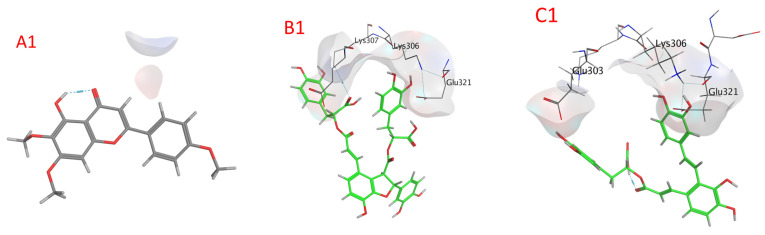

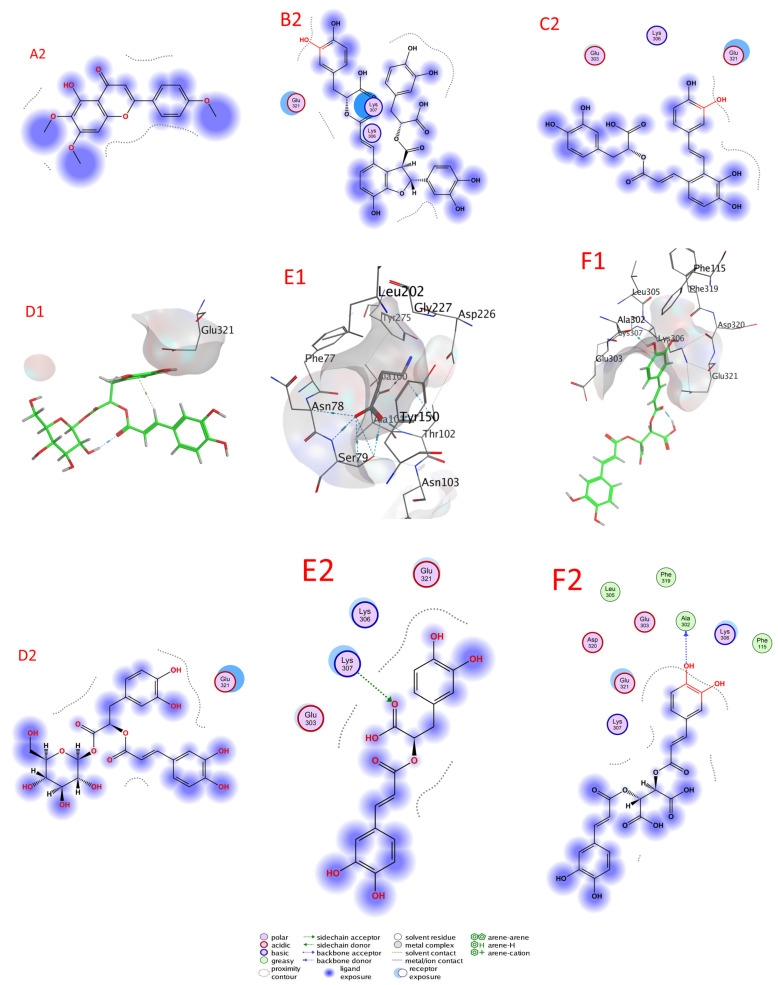
A docking image of the binding sites of gamma-aminobutyric acid–transaminase (GABA-T) (3IP9) with salvigenin (**A1**,**A2**), salvianolic acid B (**B1**,**B2**), salvianolic acid A (**C1**,**C2**), rosmarinic acid glucoside (**D1**,**D2**), rosmarinic acid (**E1**,**E2**), and chicoric acid (**F1**,**F2**); complex three-dimensional (stereoview) structures (**A1**–**F1**) and two-dimensional (**A2**–**F2**) interaction maps.

Compounds’ docking scores with GABA-T appear in Table 9 and Table S5, as well as Figure 9 and Figure S5 (3IP9). The examined compounds showed low docking energy for the target enzyme (GABA-T), with values ranging from −4.1866 (fertaric acid) to −7.1403 (salvigenin) kcal/mol, as illustrated via the docking analysis (Table 7). Compounds with docking energies of approximately −7.1403 kcal/mol had higher binding affinities than those in the lower −7.5352 kcal/mol range, including salvigenin, salvianolic acid B, salvianolic acid A, rosmarinic acid glucoside, rosmarinic acid, and chicoric acid, with values of −7.1403, −5.7478, −5.3285, −5.2018, −4.9175, and −4.8675 kcal/mol, respectively. These were followed by quercetin-3-O-rutinoside, isocitric acid, nepetoidin A, ursolic acid, nepetoidin B, caftaric acid, and fetaric acid, which again seemed to have the lowest sensitivity for the GABA-T enzyme with values of −4.8259, −4.7082, −4.4911, 4.4872, −4.3959, −4.2648, and −4.1866 kcal/mol (Table 7).

### 2.9. ADMET Data

All compounds’ HBD, HBA, LogS, LogP, BBB, PPB, CYP450, and H–HT values are included in Table S6, along with a summary of the analysis of these and other properties. Table S2 includes the LogP values of 1.228–3.343 for chicoric acid, salvigenin, nepetoidin A, nepetoidin B, rosmarinic acid, salvianolic acid B, and salvianolic acid A. Values of LogP below zero and LogS values over 10 μg/mL were seen for rosmarinic acid glucoside, quercetin-3-O-rutinoside, isocitric acid, caftaric acid, and fertaric acid. LogP values of 7.09, 3.343, and 3.335 μg/mL were observed for ursolic acid, salvianolic acid B, and salvianolic acid A, respectively. LogS values of 0.187, 11.916, and 34.08 μg/mL were obtained for ursolic acid, salvianolic acid B, and salvianolic acid A, respectively (Table S6).

The PSA values of 107.22, 107.22, 57.53, 78.13, and 132.13 for nepetoidin A, nepetoidin B, ursolic acid, salvigenin, and isocitric acid demonstrated a deficiency in their capacity to penetrate cell membranes. On the contrary, the PSA values of 144.52, 150.59, 161.59, 184.98, 223.67, 269.43, 208.12, and 278.04 for rosmarinic acid, fertaric acid, caftaric acid, salvianolic acid A, rosmarinic acid glucoside, quercetin-3-O-rutinoside, chicoric acid, and salvianolic acid B suggested effective cell membrane permeability.

CYP450 had a positive correlation to salvigenin (++), rosmarinic acid (+), salvianolic acid B (+), and rosmarinic acid glucoside (+) and a negative correlation to quercetin-3-O-rutinoside (---), nepetoidin A (-), nepetoidin B (-), ursolic acid (-), chicoric acid (-), isocitric acid (-), caftaric acid (-), fertaric acid (-), and salvianolic acid A (-). Rosmarinic acid, nepetoidins A and B, salvigenin, caftaric acid, and fertaric acid had a positive correlation with hepatotoxicity, while ursolic acid, chicoric acid, isocitric acid, salvianolic acid A, and salvianolic acid B had a negative correlation with hepatotoxicity. PPB was significantly higher in all compounds. Salvigenin (++), fertaric acid (++), rosmarinic acid (+), nepetoidin A (+), nepetoidin B (+), and caftaric acid (+) had positive H–HT relationships, whereas ursolic acid (---), quercetin-3-O-rutinoside (---), salvianolic acid B (---), chicoric acid (-), isocitric acid (-), and salvianolic acid A (-) had negative H–HT relationships.

## 3. Discussion

Optimal growth conditions for *O. basilicum* were determined by optimizing cell and tissue cultures starting with callus induction. This study demonstrated that the concentration and content of plant growth regulators (PGRs) had a significant impact on the rate of callus formation. The lack of 2,4-D and KT in the medium resulted in the moderate production of callus frequencies. The majority of fawn and buff calluses exhibited an increased tendency to develop into somatic embryos when treated with a combination of PGR 2,4-D at concentrations of 0.1 mg/L and 0.5 mg/L kinetin. The results indicated that the MS medium supplemented with 2,4-D and kinetin had the most significant effect on the growth of *O. basilicum* callus. The preceding ratio indicated that there was an 84.7% initiation rate when using 1 mg/L IBA. Additionally, the number of callus forms reduced while using IAA at a concentration of 1 mg/L. After 40 days of therapy, the callus underwent a transformation and became an embryogenic callus. The presence of elevated levels of NAA and kinetin resulted in the failure of most of the transplanted calluses to form shapeless calluses, exhibited decreased growth, and did not lead to the development of any embryos, which may have inhibitory effects when present in greater quantities. Upon examining the additives used in the altered MS medium, it was found that sucrose, when present at a concentration of 3%, exhibited superior performance compared to glucose. The optimal concentration of plant growth regulators (PGRs) for initiation was 0.5, 0.5, 0.1, and 1 mg/L of 2,4-D, kinetin, NAA, and IBA, respectively. The LS liquid medium consistently produced a higher number of somatic embryos. The cell suspension of *O. basilicum* saw a significant increase in weight after infection with V. dahliae. The weight values increased progressively as the callus aged and was infected with *V. dahliae*.

The current result confirms previous findings by Açıkgöz [34] and Kintzios et al. [72], who also established the effectiveness of NAA and kinetin supplementation in promoting suspension culture growth in the same *Ocimum* species. Exponential growth in the biomass yield of the currently established *O. basilicum* suspension culture was observed starting from the 30th day of cultivation in the optimized medium. The maximum growth phase was reached on the 40th day with biomass yields of 7.98 and 6.63 g/200 mL of medium with and without *V. dahliae* infection, respectively (Table S1). Prior data further support the current pattern of biomass buildup despite variations in the kinds and amounts of plant growth regulators [73,74]. Regarding the production of secondary metabolites in shake flasks, it is worth mentioning that a significant number of polyphenolic compounds can be obtained in the established suspension culture of *O. basilicum*. This culture also leads to the accumulation of various flavonoids and phenolics, including rosmarinic acid, ursolic acid, nepetoidin A and B, salvigenin, and quercetin-3-O-rutinoside. This information is supported by the UPLC-I TQD MS analysis, as shown in Table 5 and Figure S1. The enhanced biosynthetic capacity of the suspension culture compared to calluses may be attributed to its superior ability to efficiently absorb nutrients and oxygen in the liquid medium, which provides optimal growth circumstances, as previously explained [74,75].

*Ocimum basilicum*’s secondary metabolites were investigated, revealing considerable insecticidal activity against adult *R. ferrugineus* using chicoric acid, ursolic acid, salvigenin, quercetin-3-O-rutinoside, salvianolic acid B, rosmarinyl glucoside, salvianolic acid A, and nepetoidin B. Other compounds showed the highest topical application effectiveness against larvae, i.e., chicoric acid, salvigenin, salvianolic acid B, salvianolic acid A, nepetoidin B, and rosmarinic acid. Extracts of *O. basilicum* showed efficacy, with particular action against AChE, ALPs, ACPs, and GABA-T in vitro and in vivo. The flavonoids, polyphenolic acids, and related secondary metabolite synthesis were dramatically increased in cell suspensions, especially those infected with *V. dahliae*. However, reduced feeding in larvae was observed, demonstrating its efficacy and effectiveness. Adults, however, were the most likely to notice its antifeedant effect.

Our findings suggested that the highest incidence of antifeedant action occurs under optimal conditions. It is possible that the large variety of bioactive metabolites discovered in the extract contributed to its potent insecticidal activity. In the *O. basilicum* cell suspensions, their amounts of flavonoids and phenolic acids climbed slowly over the first 30 days of initiation before exploding and remaining high during the final 10 days. For substances tested with an IC_50_ rate, dose differences had a considerable impact. The effect of *O. basilicum* may be understood in terms of *R. ferrugineus* fourth-instar larvae’s midgut under chemical stress extracted in vitro, revealing AChE, ACP, ALP, and GABA-T activity. Insecticidal action against *R. ferrugineus* AChE, ACP, ALP, and GABA-T enzymes was explained, and it demonstrated the activity of basil extract according to these chemical components. As these extracts contain antifeedant compounds, it is also unknown which insects they target and how they work [76,77]. Based on the findings, potential pathways behind the insecticidal action include feeding physiology disruption, persistent toxicity, and repellency [78]. Major precursors to flavonoids and polyphenolic acids were found in extracts, making flavonoids and polyphenolic acids the most important secondary metabolites [79,80,81]. As these secondary molecules grow on cells, they provide an essential defense against infections; their importance has recently been underlined [82,83,84]. Therefore, cutting-edge methods such as the green biosynthesis of biologically active secondary metabolites are in high demand [62,85,86,87]. In addition, due to its fast responsiveness and cell division, in terms of both efficiency and speed, the cell suspension culture trumps the callus culture as a method for boosting bioactive chemical production [11,62,87,88,89,90]. Investigations showed that the secondary metabolites of *O. basilicum* extracts had varying effects on AChE, ALP, ACP, and GABA-T inhibition (in vitro and in vivo) (Figure 4 and Figure 5), demonstrating the importance of AChE, ALPs, ACPs, and GABA-T in the extracts’ mechanism of action. Evidence suggests that *O. basilicum* extract contains some compounds, including rosmarinyl glucoside, salvianolic acid A, quercetin-3-O-rutinoside, chicoric acid, salvianolic acid B, rosmarinic acid, fertaric acid, salvigenin, nepetoidin B, caftaric acid, and ursolic acid, that may be cytotoxic for AChE, ALPs, ACPs, and GABA-T with concentrations of 3.72, 0.49, 2.67, 1.32, 0.59, 15.94, 0.14, 2.86, 6.21, 0.41, and 5.12 µmol g^−1^ per cell, respectively. The data on the fourth-instar larvae midgut preparation revealed that *O. basilicum* extract inhibits AChE, ACPs, ALPs, and GABA-T in vivo, with lower values than those in vitro.

The ADMET study showed that chicoric acid, salvianolic acid B, salvianolic acid A, salvigenin, nepetoidin B, and rosmarinic acid were hydrophobic and penetrated the biological membranes. LogP values showed that the compounds quercetin-3-O-rutinoside, rosmarinyl glucoside, fertaric acid, caftaric acid, and isocitric acid all had low permeability into lipid bilayers (−1.687, −0.98, −0.144, −0.447, and −1.393, respectively). Poor aqueous solubility (LogP 7.09) characterized ursolic acid, whereas intermediate solubility (34.08 and 11.916 μg/mL) was seen for salvianolic acids B and A, according to LogP values of 3.335 and 3.343, respectively. These data suggested that chicoric acid, salvigenin, nepetoidin A, nepetoidin B, fertaric acid, caftaric acid, and rosmarinic acid had a relatively high permeability in the oral route in accordance with the Lipinski criterion. Chicoric acid, salvigenin, salvianolic acid B, salvianolic acid A, nepetoidin A, nepetoidin B, and rosmarinic acid are all compounds that have the potential to act as bioinsecticides, but the latter’s efficacy is determined in part by how well they are absorbed, distributed, metabolized, and excreted and how toxic they are to CYP450 substrates and enzymes. Rosmarinic acid, salvigenin, rosmarinic acid glucoside, and salvianolic acid B were inhibited, while nepetoidin A, nepetoidin B, ursolic acid, chicoric acid, iso-citric acid, salvianolic acid A, and caftaric acid demonstrated no inhibition, indicating good metabolic stability. Predictions of hepatotoxicity indicated, however, that the chemicals rosmarinic acid, nepetoidin A, nepetoidin B, salvigenin, caftaric acid, and fertaric acid could harm liver cells. The docking research showed that each ligand studied targeted active pockets of AChE, ACP, ALP, and GABA-T enzymes by forming van der Waals hydrogen bonds between amino acids. According to in silico investigations [11,66], the chemicals chicoric acid, salvianolic acid B, salvianolic acid A, salvigenin, nepetoidin B, and rosmarinic acid optimally satisfy some ADMET criteria. Molecular docking is defined as an investigation of the molecular position or orientation on possible targets to estimate binding interactions. H-bonds, H–pi hydrophobic, pi–pi bonds, and van der Waals contacts were among the interactions discovered by docking compounds onto AChE, ACPs, ALPs, and GABA-T. Previously observed bond interactions have helped scientists understand the biological functions of several substances in many disciplines, including pharmaceuticals and insecticides [11,65]. The IC_50_ values for *O. basilicum* extract suggested the significant suppression of AChE, ACPs, ALPs, and GABA-T extracted from the fourth-instar larvae midgut preparation, demonstrating insecticidal efficacy against *R. ferrugineus*. These findings highlight the potential building blocks for the development of the production of secondary metabolites using green technology and cell suspension techniques.

## 4. Materials and Methods

### 4.1. Biochemicals, Media, and Reagents

Reagents, media, solvents, and biochemical substances were provided by Sigma-Aldrich Chemical Co., Mo. (St. Louis, MO, USA). All testing materials were provided by Merck Chemical Co. (St. Louis, MO, USA), Aobious Inc. (Gloucester, MA, USA), and BioCrick Biotech (Keyuan Road 88, Chengdu, China). The *V. dahliae* strain was obtained from the Pest Control Laboratory, King Faisal University, SA.

### 4.2. Plants

The seeds of *O. basilicum* were sourced from Al-Ahsa, Saudi commercial nurseries, throughout February and March. The King Faisal University Research and Training Station, Saudi Arabia, grew plants from sterile seeds for eight weeks (pH: 5.7; seedlings: 19–20 cm) by placing them at 24–28 °C for 16 h of the light in an incubator.

### 4.3. Initiation of Calluses in O. basilicum Using V. dahliae and a Combination of Plant Growth Regulators (PGRs)

Seeds germinated in a Petri plate coated with the sterile blotting paper at 25 ± 3 °C and intensities of 28 Einsteins/(m^2^·s). Several quantities of abiotic elicitors were added to MS media containing *O. basilicum* (explants, such as cotyledonary, epicotyls, and hypocotyls with lengths of 4–5 mm) supplemented with variant values as mg/L of PGRs (such as the following: 0.1, 0.5, and 1 of kinetin or 2,4-D or IAA; 0.01, 0.1, 0.5, and 0.01, 0.1, 0.5, and 1 of NAA; 1 of BAP or IBA) and 3% (*w*/*v*) of glucose or sucrose and using experiments without PGRs as a control [11,34]. A climate room (26  ±  2 °C with light for 16 h) was used to keep all treatments viable for eight weeks, including subcultures every three weeks. This study used *V. dahliae* as an initiator to investigate the possibility of boosting callus development [11]. Calluses were extracted from respective cultures using filtration with a vacuum pump after infection for 72 h, and further visual inspections were performed every five days for the next 40 days. Subculturing occurred every 35–40 days when growing *V. dahliae* in PDA incubated at 20–22 °C.

Conidia were grown in a rotary shaker at 22 °C and 240 rotations per minute in a potato dextrose (PD) medium for ten days. The conidia were collected using centrifugation and then washed three times using 0.1 M potassium monohydrogen phosphate–potassium dihydrogen phosphate (K_2_HPO_4_-KH_2_PO_4_; pH: 6.5). Microscopically, the number of conidia was counted using a hemocytometer. Finally, 10 mL of a fresh MS solid medium was injected with 30 µL of a *V. dahliae* suspension containing (3–5) × 10^7^ conidia.mL^−1^ or purified sterile water used as a control.

### 4.4. Cell Suspension O. basilicum

The initiation of callus formation in the LS media was observed over five weeks. Several sizes of screens were used to filter the medium [11,21,34]. After filtering and adding 25 mL of conidial *V. dahliae* suspension containing (3–5) × 10^7^ conidia.mL^−1^ to the LS medium (200 mL), the mixture was adjusted to 250 mL in Erlenmeyer flasks (500 mL) using the LS medium. The protein content was determined after 72 h of culture collection. Conical flasks were seeded with a suspension culture in the LS medium (100 mL in a 250 mL flask). An incubator was used to maintain the cells at 30 ± 2 °C with light for 16 h and a rapid shaker rate at 110 rpm for six weeks before they were subcultured every two weeks.

### 4.5. Evaluation of Cell Suspension and Callus Content

Harvesting cultures for phenolic content detection began 72 h after inoculation, and cell suspensions were performed at 5-day intervals for 5–40 days or until the callus reached 40 days of age.

#### 4.5.1. Analysis of Total Phenolic Content (TPC)

The TPC (in the callus or cell suspension) was identified after 12 h of methanol extraction [74] with shaking and subsequent anhydrous Na_2_SO_4_ drying. The test solution (10 µL) and Folin–Ciocalteu solution (50 µL) were applied to 790 µL of distilled water [91]. The mixture underwent a vortex to break up any large particles, 150 µL of 20% Na_2_CO_3_ (*w*/*v*) was added for 1 min, and then the mixture was left to incubate at 25 °C for 120 min (in the dark). Phenolic acid was measured at 750 nm using a Shimadzu UV mini-UV–VIS spectrophotometer (Shimadzu Corporation, Kyoto, Japan) with an approximation made from a gallic acid reference curve.

#### 4.5.2. Analysis of Flavonoid Content (TF)

Extract testing began with a 500 µL extraction of a mixture containing the extract and 5% sodium nitrate (*w*/*v*), followed by observation of the resulting solution and then the addition of 10% AlCl_3_ (*w*/*v*) (300 µL) from samples (cell suspension and callus) [91]. Following five minutes of room-temperature incubation, NaOH (1 mL, 1M) was inserted to stop the reaction. The entire amount of flavonoid was calculated using 510 nm spectroscopy (Shimadzu UV mini-UV–VIS spectrophotometer), and the amount was computed using a standard curve of quercetin with concentrations of 1–100 mg (100 g^−1^ per DW).

### 4.6. LC-MS Analysis

We used a UPLC-I-class Waters Acquity combined with Xevo TQD MS (Waters Corporation, 34 Maple Street, Milford, MA, USA) [51,55] to determine variations in the polyphenolic acid and flavonoid content in extracts from the *O. basilicum* cell suspensions. After 30 min of shaking at 4 °C with 5 mL of methanolic extract, the extraction process included a 5-min sonication period followed by 10 min of centrifugation at 3000× *g*. The supernatant was collected and filtered using 0.45 m of polyvinylidene difluoride (PVDF) to determine the MW and chemical similarity of molecules. The extracted ion current (EIC) from the MS spectra was measured and found to be 40-ppm-efficient. In the process of injecting 5 μL of sample solution, 2 μL:1 mL of n-hexane was added to the auto-sampler injectors. With solvents A (a solution of 1% of formic acid (*v*/*v*) in distilled water) and B (as acetonitrile), the gradient was as follows: gradient elution (solvent B, 10–20% in 20 min, 20–25% in 10 min, and 25–30% in 10 min) followed by a 10-min period of gradient elution at an 8 mL/min flow rate; a pressure of 60 psi for nitrogen and 7 psi for argon in the collision cell; Acquity UPLC BEH type C18 with a 1.7 μm–2.1100 mm column and 0.5 mL/min as the flow rate, and an injection capacity of 10 L using the software and mass library Masslynx v4.1 and UPLC-I class Waters Acquity connected with Xevo TQD MS. The electrospray ionization (ESI) was set up as the air pressure source, allowing the MS to function in negative ion modes. It had a vaporizer flow rate of approximately 12 L/h. After that, the gas was heated to 300 °C, and the voltage across the electron impact capillary was raised to 3000 V (dry state). Information was collected using mass spectrometry in scan mode (*m*/*z* 100–900). The MS spectra were used to quantify the compounds by first obtaining the molecular weight and structural similarity, and the EIC was compared to a relatively high proportion calibration graph. Three duplicates of each condition were subjected to quantification, with the results verified using Xevo TQD MS and the Acquity UPLC-I class assignment.

### 4.7. Analyzing the Activity as an Antifeedant and Contact Insecticide against R. ferrugineus Using Separated Secondary Metabolites

Over the course of 40 days, an *O. basilicum* cell suspension was cultivated in this investigation, and then the extract was obtained. Additionally, compounds of ursolic acid, rosmarinic acid, isocitric acid, chicoric acid, caftaric acid, fertaric acid, nepetoidin B, salvigenin, rosmarinyl glucoside, quercetin-3-O-rutinoside, salvianolic acid A, and salvianolic acid B were created with acetone at concentrations of 1, 10, 50, 100, 500, 1000, 2000, and 3000 µg/mL, with the final volume being built up with 0.1% (*v*/*v*) TritonX-100 [21,92]. Long segments of sugarcane stem were employed in the insect breeding lab at King Faisal University’s Research and Training Center to raise adult *R. ferrugineus* larvae. For this experiment, we used a topical application technique with larvae stored at 4 °C for five minutes to test the extract’s activity. Using a hand-operated micro-applicator (Burkard Manufacturing Co., Ltd., Hertfordshire, UK, collected using a 50 ll micro-syringe), extracts at escalating ratios (10 µL) were injected into the dorsal surface of the larvae (Ito Corp.; MS-N50, Shizuoka, Japan). Five larvae per box in a series of three replicates were fed on sugarcane stem pieces measuring 10 cm in length. The LD_50_ was calculated by measuring the percentage of dead larvae at 24, 48, 72, and 96 h following a topical application. The antifeedant impact was determined in adults by giving them 10 cm sugarcane stem segments cut in half longitudinally. Long strips of sugarcane stem (32 cm^2^) were immersed in the previous serial extract and compound concentration (10 mL) for ten seconds before they were dried in ambient air using a plastic box. Each box included a new pair of larvae (a male and a female). Ten sets of each treatment were performed. Data on how well the subjects ate were evaluated at 24, 48, 72, and 96 h.

### 4.8. Protein Measurement of R. ferrugineus Larvae from the Fourth Instar

The *R. ferrugineus* were homogenized larvae in a buffer (sodium phosphate, 40 mM, and pH 7.4); then, sodium chloride (10 mM dissolved in 1% (*w*/*v*) triton X-100) was added. During centrifugation (Sigma 3–30 KS), the homogenate was chilled to 4 °C and spun at a speed of 5000 rpm (20 min). The supernatant was either immediately put into action for an enzymatic test or frozen and kept at 20 °C for later use. Each test was repeated three times. The protein content was determined using the same protocol employed by Lowry with modification [93] using protein extract (100 µL) combined with an alkaline copper solution and a Folin–Ciocalteu phenol reagent (2 mL and 200 µL, respectively). The intensity of the resulting blue hue was evaluated during 30 min of incubation at 25 °C. Using an ELISA plate reader and a BSA standard curve, we could calculate the protein concentration by analyzing the absorbance at 600 nm (Multiskan SkyHigh Microplate Spectrophotometer, Thermo Fisher Scientific, Waltham, MA, USA).

### 4.9. In Vitro AChE Activity in R. ferrugineus Fourth-Instar Larvae

The enzyme AChE was assessed using a substrate of acetylthiocholine iodide and evaluated using 5,5′-dithiobis [2-nitrbenzoic acid] (DTNB), according to [94]. Each well contained a sodium phosphate buffer (140 µL) (pH: 8.0; 100 mM), DTNB (10 µL), extract (20 µL)/components at 1, 10, 50, 100, and 1000 µg/mL in DMSO and underwent 15 min of incubation at 25 °C. Acetylthiocholine (ATCh) was added to start the reaction (10 µL). The ATCh enzymatic hydrolysis produced a 5-thio-2-nitrobenzoate anion as a color (yellow) that was detected by measuring its wavelength, i.e., 412 nm, thereby allowing the researchers to track the rate of ATCh hydrolysis (15 min). Each response was performed three times on a microplate and examined using an ELISA plate reader to determine the 412 nm absorbance of the combination (Multiskan SkyHigh Microplate Spectrophotometer). The AChE-specific activity was measured as OD412/mg protein.h. based on observing the effect of raising the quantities of all of these compounds during the tests on the inhibition results, and the inhibitory concentration (IC_50_) was calculated. Then, a computer algorithm was used to determine the IC_50_ values (Version 9.01 of GraphPad Prism by Inc., 2020, San Diego, CA, USA).

### 4.10. In Vitro ACP and ALP Phosphatase Activity in R. ferrugineus Fourth-Instar Larvae

Activities of ACPs and ALPs were evaluated using a modified version of the Asakura technique [95]. The ACP test required incubating 100 μL of crude enzyme in a buffer of 650 μL of sodium acetate (pH: 5.0; 50 mM with ACPs) or 250 μL of Tris–HCl (at 50 mM; pH: 8.0 with ALPs) and p-nitrophenyl phosphate (5.5 mM). For 30 min; the various mixes were kept at 37 °C for incubation. Thereafter, 500 μL of NaOH (0.5 N) stopped the reaction. Using an ELISA plate reader, we determined the mixture’s 415 nm absorbance value (Multiskan SkyHigh Microplate Spectrophotometer). The ACP- and ALP-specific activity was estimated as OD 415/mg protein·h.

### 4.11. In Vitro GABA-T Activity in R. ferrugineus Fourth-Instar Larvae

The GABA-T activity was assessed with a modified version of the method published by [96]. The enzyme crude extract (25 μL) added to a solution included Tris-HCl, α-ketoglutarate, 2-mercaptoethanol, β-NAD, and GABA (50, 2, 20, and 3, respectively) at a volume of 1525 μL. Finally, 30 μL of tritonx100 ((1%, (*v*/*v*)) was added to the prior combination. We used 30 min of incubation at between 25 and 30 °C. Additionally, we used an ELISA plate reader to determine the absorbance of the combination at 340 nm (Multiskan SkyHigh Microplate Spectrophotometer). Specific activities of GABA-T were measured as OD 340/mg of protein·h.

The methanol extract or purified chemicals (rosmarinic acid, ursolic acid, isocitric acid, chicoric acid, caftaric acid, fertaric acid, salvianolic acid A, and salvianolic acid B, nepetoidin B, salvigenin, rosmarinyl glucoside, and quercetin-3-O-rutinoside) at 1, 10, 50, 100, 500, 1000, 2000, and 3000 mg/L were tested on fourth-instar larvae of *R. ferrugineus* for 24 h. Each enzyme’s in vivo impact was measured after treating larvae with an extract or pure chemicals. An inhibitor-free test combination served as a negative control. We determined the absorbance of the enzymes throughout a spectrum of wavelengths using a rapid reader for ELISA plates (Multiskan SkyHigh Microplate Spectrophotometer). Extracts and pure chemicals were used to treat *R. ferrugineus* larvae at previous serial concentrations, and their AChE, ACP, ALP, and GABA-T activity was tested in vivo using the methods outlined above. Larvae spent a day on leaf disks treated with chemicals or extract.

### 4.12. Compound Docking with Analyzed Enzymes

Including the Protein Data Bank (PDB), we retrieved the AChE (PDB:1QON) [97] ACP (PDB:3IT3) [98], ALP (PDB:2B0C) [99], and GABA-T (PDB:3IP9) [100] structures and imported them into the Molecular Operating Environment (MOE). Crystallographic water molecules and heteroatoms were removed to correct the chemical changes in the protein caused by the absence of hydrogen [101]. Compounds were generated using ChemDraw Pro (module 15, Builder). The ligands were fine-tuned, a three-dimensional structure was constructed, duplicates were removed, and new bonds were introduced before docking. The ligands were then fine-tuned and meticulously put into the enzyme modeling catalytic site cavity after using the default values and determining the lowest-energy structures. To use an allosteric approach, presuming that the receptor was set and the ligand was variable, docking was performed using MOE 2015.10 (Chemical Computing Group Inc., Montreal, QC, Canada). To calculate the binding affinity, a full squad was applied. The ligand molecules and protein affinities were calculated using score algorithms that measured the energy of non-covalent (free-binding) interactions in molecular protective shield parlance. After the docking process, the processes generated and RMSD were computed, and the results were examined and discussed to determine the best possible ligand molecule–protein interaction.

### 4.13. ADMET Monitoring

To evaluate the potential toxicity of the pure chemicals under study, ADMETLAB 2.0 was used for the ADMET evaluation. ADMET characteristics were predicted for every compound based on its water solubility, LogS, intestinal absorption, LogP, HBA, HBD, BBB, PPB, H–HT, and CYP450. Many empirical data sources were used to create the models used in this technique, and their origins and descriptions can be found in the accompanying documentation.

### 4.14. Statistical Design

SPSS 25.0 was used for the statistical analysis, which included a probit analysis in accordance with Finney [102]. The tissue culture parameters were calculated quantitatively from three independent sets of samples, and the mean SD was used to describe the findings. Toxicity parameters were determined quantitatively using a 10-replicate sample, and means and standard deviations were used to present the data. The median dosage obtained from a dose–response regression was used to calculate the LC_50_ value. By analyzing the comparative growth rate vs. the crude extract logarithm using least squares regression, the range of LC_50_ was calculated with 95% confidence. A simple variance study was performed on the increased weight and enzymatic activity data (ANOVA). The distinction between the means was accomplished using the Student–Newman–Keuls (SNK) test. Additionally, SPSS version 25 was used to find *p* < 0.05 significant deviations from the mean.

## 5. Conclusions

The ongoing cultivation of an *O. basilicum* cell suspension culture revealed its previously undiscovered capacity for producing various insecticidal compounds. These compounds are relevant to agricultural and food industries, antimicrobial packaging, and the pharmaceutical sector, specifically in the production of highly sought-after flavonoids and phenolics. This indicates the unique preference of this culture for synthesizing these compounds. Furthermore, the already-perfected approach of cultivating cell suspensions has significant practical advantages for commercial use, as it allows for a more dependable and predictable method of accumulating bioactive products. The strategic utilization of the elicitation- and bioreactor-based cultivation process has the potential to significantly enhance the yield of *O. basilicum* in vitro technology. This, in turn, can meet the global demand for bioactive phenolic and flavonoid compounds, ensuring long-term industrial sustainability. These secondary metabolites detected from *O. basilicum* extract show potential as bio-insecticides to the red palm weevil. In particular, the phenolic compounds in *O. basilicum* seem to be responsible for the observed correlation between secondary metabolites and their applications. The importance of this study can be used to evaluate the benefits of using secondary metabolites for future potential applications as eco-friendly biopesticides. Furthermore, a simple and hygienic approach (cell suspension) allowed us to interpret the massive-scale generation of such secondary metabolites, which might then be used as biomarkers for resistance and plant protection.

## Figures and Tables

**Table 1:** Frequency of callus induction and callus status with KT, 2,4-D, IAA, and IBA from *O. basilicum*.

Treatment and Value	*O. basilicum*				
	KD1	KD2	KD3	KD4	KD5
Initiation ratio (%) with IBA (1)	5.96 ± 0.15 ^d^	84.66 ± 0.58 ^a^	60.66 ± 1.53 ^b^	44.66 ± 0.57 ^c^	6.03 ± 0.152 ^d^
Initiation ratio (%) with IAA (0.1)	7.66 ± 0.57 ^e^	83.33 ± 1.53 ^a^	54.66 ± 1.53 ^b^	26.33 ± 1.52 ^d^	43.66 ± 1.53 ^c^
Callus net weight (g/40 d) with IBA	0.8866 ± 0.0152 ^c^	4.88 ± 0.025 ^a^	3.12 ± 0.02 ^b^	3.94 ± 0.031 ^ab^	0.97 ± 0.02 ^c^
Callus net weight (g/40 d) with IAA	0.72 ± 0.015 ^c^	4.52 ± 0.025 ^a^	3.036 ± 0.030 ^b^	0.152 ± 0.02 ^d^	0.95 ± 0.015 ^c^

PGRs: plant growth regulators; concentration of PGRs in mg/L; KT: kinetin; 2,4-D: 2,4-Dichlorophenoxyacetic acid; IAA: indole-3-acetic acid; IBA: indole-3-butryic acid; KD1 = KT, 2,4-D (1); KD2 = KT (0.5), 2,4-D (0.1); KD3 = KT, 2,4-D (0.1); KD4 = 2,4-D (0.1); KD5 = KT (0.1), 2,4-D; means with similar letters were not statistically different at a significance level of 0.005, as determined via the LSD test; data analysis performed with a one-way analysis of variance (ANOVA).

**Table 2:** Frequency of callus initiation and callus strength with KT, NAA, 2,4-D, BAP, glucose, and sucrose from *O. basilicum*.

Treatment and Value	KDN1	KDN2	KDN3	KDN4	KDN5
% initiation with 3% sucrose (*w*/*v*)	61.33 ± 1.53 ^b^	80.66 ± 1.53 ^a^	52.66 ± 1.53 ^c^	27.66 ± 1.52 ^d^	7.33 ± 0.58 ^e^
% initiation with 3% glucose (*w*/*v*)	50.33 ± 2.08 ^b^	70.66 ± 1.154 ^a^	48 ± 1.0 ^c^	24.33 ± 0.57 ^d^	5.66 ± 0.58 ^e^
**Treatment and Value**	**KDB1**	**KDB2**	**KDB3**	**KDB4**	**KDB5**
% initiation with 3% sucrose (*w*/*v*)	41.66 ± 0.57 ^b^	72.0 ± 1.0 ^a^	42.66 ± 0.57 ^b^	21.0 ± 1.0 ^c^	4.66 ± 0.58 ^d^
% initiation with 3% glucose (*w*/*v*)	32.66 ± 1.52 ^c^	57.33 ± 1.53 ^a^	38.66 ± 1.53 ^b^	14.66 ± 1.53 ^d^	3.33 ± 0.577 ^e^

Concentration of PGRs in mg/L; 2,4-D: 4-Dichlorophenoxyacetic acid; KT: kinetin; NAA: 1-naphthylacetic acid; IBA: indole-3-butryic acid; BAP: 6-Benzylaminopurine; KDN1 = KT, 2,4-D (1), NAA (0.01); KDN2 = KT (0.5), 2,4-D, NAA (0.1); KDN3 = KT, NAA, 2,4-D (0.1); KDN4 = 2,4-D (0.1), NAA (0.5); KDN5 = KT (0.1), NAA (1); KDB1 = KT, BAP (1), 2,4-D (0.5); KDB2 = KT, BAP, 2,4-D (0.5); KDB3 = KT (0.1), BAP, 2,4-D (0.5); KDB4 = 2,4-D (0.5), BAP (0.2); KDB5 = KT, BAP (0.1), 2,4-D (0.5); means with similar letters were not statistically different at a significance level of 0.05, as determined via the LSD test; data analysis performed with a one-way analysis of variance (ANOVA).

**Table 3:** Callus induction frequencies and callus status with the best PGRs and chemicals from *O. basilicum*.

Value	*O. basilicum*
KT	0.5
2,4-D	0.1
IAA	0
IBA	1
NAA	0.1
Sucrose (% *w*/*v*)	3
% initiation	100
Callus net weight (g/40 d) without *V. dahliae*	7.11
Callus net weight (g/40 d) with *V. dahliae*	10.51
Color	Brown
Texture	Hardy

Concentration of PGRs in mg/L; 2,4-D: 4-Dichlorophenoxyacetic acid; KT: kinetin; NAA: 1-naphthylacetic acid; IBA: indole-3-butryic acid; IAA: indole-3-acetic acid; data analysis performed with a one-way analysis of variance (ANOVA).

**Table 4:** Chemical analyses of cell-suspension-extracted *O. basilicum* and calluses infected and uninfected with *V. dahlia*.

Callus or Cell Suspension	TPC (Gallic Acid As mg Per g DW)	TFC (Quercetin mg Per g DW)
Callus without infection	12.48 ± 0.1364	1.47 ± 0.0482
Callus with infection	21.75 ± 0.1872	1.95 ± 0.0634
Cell suspension without infection	19.31 ± 0.1547	2.97 ± 0.0754
Cell suspension with infection	39.68 ± 0.2451	5.49 ± 0.0941

Total flavonoid content (TFC), total phenolic content (TPC), and mean value with or without a standard deviation (SD) with a sample size of three, based on a Student–Newman–Keuls (SNK) test.

**Table 5:** Composition of secondary metabolites identified in *O. basilicum* cell suspensions using UPLC–I Class compiled with Xevo TQD MS (negative mode).

No.	Tentative Compounds	RT (min)	RI (exp)	Formula	[M − H]^−^ (*m*/*z*)	Fragmentation Ions (*m*/*z*)	Conc. (µmol g^−1^ Cell)
1	Tartaric acid	1.02	1249	C_4_H_5_O_6_	149.00	149, 141, 131, 113, 103, 87	0.19
2	Isocitric acid	2.53	1805.4	C_6_H_7_O_7_	191.0175	191, 173, 129, 111	1.62
3	Caffeic acid derivative	4.11	2155	C_18_H_32_O_4_Si_3_	359.70	396, 381, 359, 219, 191, 75	0.30
4	Caftaric acid	5.62	2701.3	C_13_H_12_O_9_	311.04	311, 179.03, 149.01, 135.04	0.41
5	Caffeic acid	6.25	1854.3	C_9_H_8_O_4_	179.03	179, 135	0.27
6	Fertaric acid	6.30	5191.1	C_14_H_14_O_9_	325.06	325, 193, 134	0.14
7	Salvianolic acid H/I	6.36	5237.8	C_27_H_22_O_12_	537.10	537, 493, 339, 313, 295, 197, 179	0.30
8	Salvianolic acid K	9.55	4556.9	C_27_H_24_O_13_	555.11	555, 537, 493, 295	0.25
9	Chicoric acid	11.16	4552.3	C_22_H_18_O_12_	473.07	473, 311, 293, 179, 149	1.32
10	Lithospermic acid	11.29	4920.2	C_27_H_22_O_12_	537.10	537, 493, 356, 295	0.41
11	Dihydroquercetin 3-glucoside	11.45	4505.7	C_21_H_22_O_12_	456.10	467, 465, 313, 285, 259, 256, 175, 151	0.029
12	Quercetin-3-O-rutinoside	11.50	4992.3	C_27_H_30_O_16_	611.16	611, 465, 449, 303	2.67
13	Rosmarinic acid	11.58	3504.5	C_18_H_16_O_8_	359.08	359, 197, 179, 161, 135, 117	15.94
14	Salvianolic acid E	12.67	4627.5	C_36_H_30_O_16_	717.15	717, 519, 475, 339	0.16
15	Salvianolic acid A	12.48	4585.8	C_26_H_22_O_10_	493.11	493, 313, 295, 185	0.49
16	Salvianolic acid B	12.59	5377.7	C_36_H_30_O_16_	717.15	717, 519, 321	0.59
17	Salvianolic acid F	17.91	4566.3	C_17_H_14_O_6_	313.07	313, 269	0.27
18	Cyanidin 3,3′-diglucoside	18.13	6158.2	C_27_H_31_O_16_	611.16	611, 287	0.028
19	Cyanidin 3-O-rutinoside	18.26	5192.3	C_27_H_31_O_15_	595.17	595, 287	0.032
20	Salvigenin	18.26	3121.7	C_18_H_16_O_6_	327.21	327, 311, 277, 215, 205, 116.9	2.86
21	Naringenin 7-0-glucoside	18.35	4081.3	C_21_H_22_O_10_	434.4	435, 271, 151, 119	0.14
22	Apigenin 7-O-glucoside	18.43	4142.7	C_21_H_20_O_10_	432.4	432, 271, 171, 147, 119	0.09
23	Rosmarinic acid glucoside A	21.35	4023.4	C_24_H_26_O_13_	521.12	359, 197, 179, 161, 135	1.97
24	Rosmarinic acid glucoside B	25.12	4061.4	C_24_H_26_O_13_	521.12	359, 323, 197, 179, 161, 135	1.75
25	Nepetoidin A	25.51	4413.7	C_17_H_14_O_6_	314.29	335, 313, 161, 133	7.35
26	Nepetoidin B	25.64	4418.9	C_17_H_14_O_6_	314.29	335, 313, 269, 161, 133	6.21
27	Ursolic acid	26.03	3658.3	C_30_H_48_O_3_	456.7	591, 524, 523, 459, 455	5.12
28	Nepetoidin glucoside	27.93	4341	C_23_H_24_O_11_	475.12	475, 323, 313, 161, 151	1.31

Data were analyzed by taking the mean (n = 3) plus the SD of three independent measurements. Fragment ion abundances were listed with their average relative abundances in brackets (z), time to retention, abbreviated RT, when a negative ion (*m*/*z*: mass/charge) was found in a molecular ion; the abbreviation was “[MH]”, determining the relative retention index, MS library relative retention index (Wiley), and NIST retention index (NIST) abbreviated (exp RI).

**Table 6:** Mortality of *O. basilicum* extract, compounds with adults and fourth-instar larvae of *R. ferrugineus* using probit analyses.

Extract and Test Compounds	Adult				Fourth-Instar Larvae			
	LC_50_ (µg/mL)	Slope	Chi-Square	*p*	LD_50_ (µg/Larvae)	Slope	Chi-Square	*p*
*O. basilicum* extract	1197 (1086–1291)	2.87 ± 0.21	49.74	0.004	12.5 (12.1–13.9)	1.43 ± 0.23	42.39	0.002
Isocitric acid	1795 (1714–1879)	2.36 ± 0.18	49.29	0.007	22.7 (21.4–23.8)	1.02 ± 0.21	39.68	0.008
Fertaric acid	1527 (1468–1617)	2.64 ± 0.19	48.76	0.005	19.46 (18.23–20.76)	1.24 ± 0.24	39.78	0.008
Caftaric acid	1492 (1382–1546)	2.76 ± 0.20	48.57	0.004	18.79 (17.82–19.93)	1.26 ± 0.23	40.02	0.007
Rosmarinic acid	1427 (1318–1489)	2.93 ± 0.21	42.61	0.006	11.8 (11.2–12.5)	1.62 ± 0.24	42.45	0.004
Nepetoidin B	1273 (1227–1314)	3.17 ± 0.20	43.29	0.003	11.5 (10.9–12.1)	1.68 ± 0.23	43.36	0.003
Salvianolic acid A	1261 (1197–1315)	3.07 ± 0.22	43.18	0.003	11.3 (10.2–12.47)	1.68 ± 0.21	43.89	0.003
Rosmarinyl glucoside	1228 (1116–1294)	3.42 ± 0.25	40.85	0.003	17.2 (15.7–18.1)	1.29 ± 0.28	40.37	0.003
Salvianolic acid B	1195 (1121–1278)	3.18 ± 0.23	42.37	0.003	11.1 (10.1–12.18)	1.61 ± 0.20	44.52	0.003
Quercetin-3-O-rutinoside	1168 (1121–1207)	3.24 ± 0.24	42.36	0.003	16.2 (15.3–17.2)	1.36 ± 0.27	39.64	0.002
Salvigenin	1143 (1067–1206)	3.20 ± 0.25	44.27	0.005	10.8 (10.2–11.5)	1.87 ± 0.22	43.82	0.003
Ursolic acid	1121 (1015–1213)	3.19 ± 0.24	43.85	0.003	14.9 (13.6–15.4)	1.36 ± 0.26	39.71	0.004
Chicoric acid	1097 (1016–1175)	3.49 ± 0.24	44.17	0.003	9.45 (9.07–10.45)	1.84 ± 0.22	46.43	0.002

Confidence limits (CF), lethal concentration (LC_50_), and lethal dosage (LD_50_); values with an SD of five replicates with each tested material; n = 10, data after 1 day of treatment; data analysis with one-way analysis of variance (ANOVA).

**Table 7:** Inhibition effect of compounds with IC_50_ for AChE, ALPs, ACPs, and GABA-T (in vitro) of fourth-instar larvae of *R. ferrugineus*.

Compounds	IC_50_ (µg/mL) In Vitro			
	AChE	ACPs	ALPs	GABA
Caftaric acid	156 ± 1.49	303 ± 3.17	461 ± 5.16	847 ± 9.52
Fertaric acid	124 ± 1.18	257 ± 2.84	394 ± 4.13	807 ± 9.14
Salvianolic acid A	94 ± 0.94	206 ± 2.65	322 ± 3.62	535 ± 6.27
Salvianolic acid B	117 ± 1.08	215 ± 2.68	319 ± 3.24	516 ± 6.07
Rosmarinic acid	119 ± 1.14	259 ± 2.81	409 ± 4.23	734 ± 8.24
Nepetoidin B	147 ± 1.38	301 ± 3.94	468 ± 4.86	842 ± 9.67
Ursolic acid	170 ± 1.52	337 ± 3.27	479 ± 4.92	848 ± 9.74
Salvigenin	130 ± 1.23	281 ± 2.90	404 ± 4.32	551 ± 6.25
Quercetin-3-O-rutinoside	98 ± 0.87	203 ± 2.14	306 ± 3.27	653 ± 7.15
Rosmarinic acid glucoside	89 ± 0.85	213 ± 2.32	333 ± 3.75	581 ± 6.34
Isocitric acid	197 ± 1.76	390 ± 4.12	584 ± 5.91	943 ± 9.82
Chicoric acid	108 ± 1.07	209 ± 2.17	339 ± 3.61	675 ± 7.41

An inhibition concentration (IC_50_) value with an SD of five replicates with each tested material; data analysis with a one-way analysis of variance (ANOVA).

**Table 8:** Inhibition effect of the compounds with IC_50_ for AChE, ALPs, ACPs, and GABA-T (in vivo) of fourth-instar larvae of *R. ferrugineus*.

Compounds	IC_50_ (µg/mL) In Vitro			
	AChE	ACPs	ALPs	GABA
Caftaric acid	429 ± 1.49	806 ± 3.17	1103 ± 5.16	1949 ± 9.52
Fertaric acid	385 ± 1.18	734 ± 2.84	1171 ± 4.13	2085 ± 9.14
Salvianolic acid A	230 ± 0.94	456 ± 2.65	670 ± 3.62	1046 ± 6.27
Salvianolic acid B	178 ± 1.08	361 ± 2.68	555 ± 3.24	880 ± 6.07
Rosmarinic acid	235 ± 1.14	494 ± 2.81	750 ± 4.23	1274 ± 8.24
Nepetoidin B	282 ± 1.38	476 ± 3.94	784 ± 4.86	1399 ± 9.67
Ursolic acid	458 ± 1.52	850 ± 3.27	1327 ± 4.92	2119 ± 9.74
Salvigenin	258 ± 1.23	553 ± 2.90	781 ± 4.32	1016 ± 6.25
Quercetin-3-O-rutinoside	348 ± 0.87	646 ± 2.14	994 ± 3.27	1702 ± 7.15
Rosmarinic acid glucoside	313 ± 0.85	632 ± 2.32	1019 ± 3.75	1683 ± 6.34
Isocitric acid	494 ± 1.76	910 ± 4.12	1358 ± 5.91	2107 ± 9.82
Chicoric acid	213 ± 1.07	417 ± 2.17	655 ± 3.61	1231 ± 7.41

An inhibition concentration (IC_50_) value with an SD of five replicates with each tested material; data analysis with a one-way analysis of variance (ANOVA).

**Table 9:** Docking values of compounds including acetylcholinesterase (AChE) (PDB:1QON), acid phosphatase (ACP) (PDB: 3IT3), alkaline phosphatase (ALP) (2B0C), and γ-aminobutyric acid-transaminase (GABA-T) (PDB:3IP9) active sites.

Compounds	Docking Value (ΔG kcal/mol)			
	AChE (1QON)	ACP (3IT3)	ALP (2B0C)	GABA (3IP9)
Chicoric acid	−8.3067	−9.4951	−7.5352	−4.8675
Ursolic acid	−6.2136	−6.2674	−7.0948	−4.4872
Salvigenin	−7.0150	−6.8416	−7.6869	−7.1403
Quercetin-3-O-rutinoside	−8.6174	−8.8978	−8.9556	−4.8259
Salvianolic acid B	−7.5902	−9.5805	−8.8400	−5.7478
Nepetoidin B	−6.3910	−6.8358	−6.2554	−4.3959
Rosmarinic acid	−7.3476	−7.0730	−6.9925	−4.9175
Caftaric acid	−6.3860	−6.9919	−6.5749	−4.2648
Fertaric acid	−7.1210	−7.3173	−7.1331	−4.1866
Isocitric acid	−4.9998	−5.4562	−5.2278	−4.7082
Nepetoidin A	−6.2567	−6.6863	−5.7638	−4.4911
Rosmarinic acid glucoside	−9.4433	−7.636	−7.7995	−5.2018
Salvianolic acid A	−9.2815	−8.3457	−8.0411	−5.3285

## Data Availability

The data presented in this study are available in the article and Supplementary Materials.

## Supplementary Materials

The following supporting information can be downloaded at: https://www.mdpi.com/article/10.3390/plants13040491/s1, Table S1. Callus and cell suspension weights from *O. basilicum* grown on synthetic solid MS and liquid LS medium at various phases of growth without or with *V. dahliae* infection. Table S2. Docking values and binding interactions of compounds with acetylcholinesterase (AChE) (PDB:1QON). Table S3. Docking values and binding interactions of compounds with acid phosphatase (ACP) (PDB: 3IT3). Table S4. Docking values and binding interactions of compounds with alkaline phosphatase (ALP) (PDB: 2B0C). Table S5. Docking values and binding interactions of compounds with alkaline phosphatase (GABA-T) (PDB: 3IP9). Table S6. Experimental chemicals’ ADMET evaluation. Figure S1. The LC-MS chromatogram from cell suspension extracts of *O. basilicum*. Figure S2. A docking image of the binding sites of Acetylcholinesterase AChE (1QON) with fertaric acid, (S2A1,2), salvigenin (S2B1,2), nepetoidin B (S2C1,2), caftaric acid (S2D1,2), nepetoidin A (S2E1,2), ursolic acid (S2F1,2), and isocitric acid (S2G1,2). Complex three-dimensional (stereoview) structures (S2A1,S2B1,S2C1,S2D1,S2E1,S2F1,S2G1,2) and two-dimensional (S2A2,S2B2,S2C2,S2D2,S2E2,S2F2,S2G2) interaction maps. Figure S3. A docking image of the binding sites of Acid phosphatase (ACP) (3IT3) with rosmarinic acid B (S3A1,2), caftaric acid (S3B1,2), salvigenin (S3C1,2), nepetoidin B (S3D1,2), nepetoidin A (S3E1,2), ursolic acid (S3F1,2), and isocitric acid (S3G1,2). Complex three-dimensional (stereoview) structures (S3A1,S3B1,S3C1,S3D1,S3E1,S3F1,S3G1) and two-dimensional (S3A2,S3B2,S3C2,S3D2,S3E2,S3F2,S3G2) interaction maps. Figure S4. A docking image of the binding sites of Alkaline phosphatase (ALP) (2B0C) with fertaric acid (S4A1,2), ursolic acid (S4B1,2), rosmarinic acid (S4C1,2), caftaric acid (S4D1,2), nepetoidin B (S4E1,2), nepetoidin A (S4F1,2), and isocitric acid (GS41,2). Complex three-dimensional (stereoview) structures (S4A1,S4B1,S4C1,S4D1,S4E1,S4F1,S4G1) and two-dimensional (S4A2,S4B2,S4C2,S4D2,S4E2,S4F2.S4G2) interaction maps. quercetin-3-O-rutinoside (S5A1,2), isocitric acid (S5B1,2), nepetoidin A (S5C1,2), ursolic acid (S5D1,2), nepetoidin B (S5E1,2), caftaric acid (S5F1,2), and fetaric acid (S5G1,2). Complex three-dimensional (stereoview) structures (S5A1,S5B1,S5C1,S5D1,S5E1,S5F1,S5G1) and two-dimensional (S5A2,S5B2,S5C2,S5D2,S5E2,S5F2,S5G2) interaction maps.
