# Supplementary material for: Exploring Ocimum basilicum’s Secondary Metabolites: Inhibition and Molecular Docking against Rhynchophorus ferrugineus for Optimal Action
Source: Plants (Basel). 2024 Feb 8;13(4):491. doi: 10.3390/plants13040491 (PMC10892409; doi:10.3390/plants13040491)
Supplement: Supplementary file 1 [file plants-13-00491-s001.zip › plants-2820239-supplementary.pdf]

Supplementary Materials

Table S1. Callus and cell suspension weights from *O. basilicum* grown on synthetic solid MS and liquid LS medium at various phases of growth without or with *V. dahliae* infection.

**Age (days)**	**Callus Weight (g) per Petri dish**		**Cell Suspension Weight (g) per 200 mL media**	
	Without Infection	With Infection	Without Infection	With Infection
**5**	2.47^{l}±0.14	3.68^{k}±0.03	2.14^{o}±0.06	2.67^{m}±0.03
**10**	3.54^{k}±0.23	4.53^{j}±0.03	2.47^{n}±0.04	3.38^{l}±0.03
**15**	4.69^{j}±0.02	6.14^{h}±0.09	3.49^{k}±0.04	4.52^{h}±0.04
**20**	5.61^{i}±0.02	6.72^{g}±0.10	3.62^{j}±0.05	5.17^{g}±0.05
**25**	6.51^{g}±0.09	8.51^{d}±0.11	4.45^{i}±0.06	5.61^{f}±0.06
**30**	7.14^{f}±0.10	9.24^{c}±0.13	5.54^{f}±0.05	6.73^{c}±0.06
**35**	7.79^{e}±0.01	10.22^{b}±0.15	5.98^{e}±0.06	7.59^{b}±0.07
**40**	8.94^{d}±0.10	11.29^{a}±0.17	6.63^{d}±0.08	7.98^{a}±0.08
F	2028		2700	
Df	15.32		15,32	

Numbers represent means standard deviations from three independent tests. The Student-Newman-Keuls (SNK) test shows that values in the same column that are headed by the same letter are not statistically different (P ≤ 0.0001 V.H.Sig. for all values). df = degrees of freedom; and F = F-ratio.

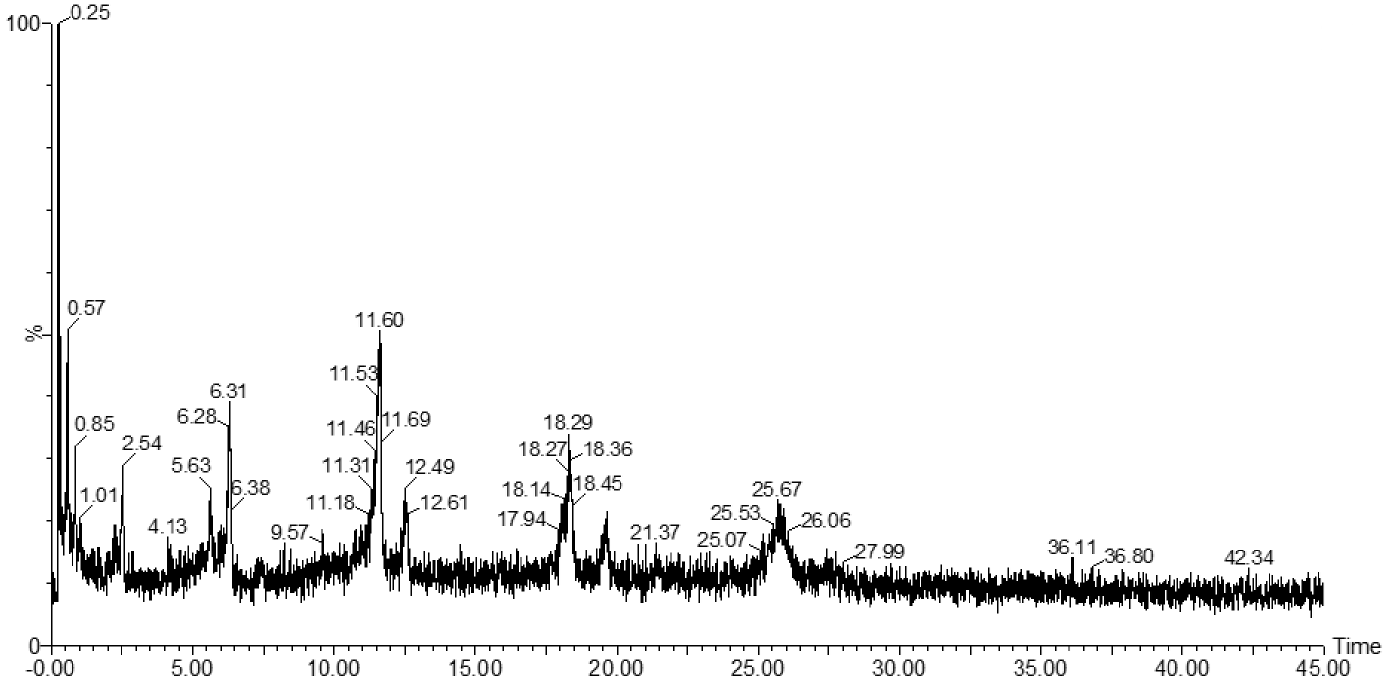

Figure S1. The LC-MS chromatogram from cell suspension extracts of *O. basilicum*.

Table S2. Docking values and binding interactions of compounds with acetylcholinesterase (AChE) (PDB:1QON)

Compounds	ΔG (kcal/mol)	H-bond (donor-Acceptor)			H-pi or pi-pi bond			van der Waals
		Ligand A. A.	interaction	Distance (Å)	Ligand A.A	interaction	Distance (Å)	
Chicoric acid	-8.3067	Ser 2085	H-acceptor	3.21	Trp 83<br>Tyr 337	pi-pi<br>pi-pi	3.93<br>3.95	Tyr 71, Gly 79, Glu 80, Gly 116, Gly 117, Thr 121, Gly 122, Leu 126, Tyr 291, Phe 297, Tyr 341, Asp 342, Trp 439, Leu 446, His 447, Gly 448, and Ile 451
Quercetin-3-O-rutinoside	-8.6174	Glu 204<br>Glu 204<br>Gly 117	H-doner<br>H-doner<br>H-acceptor	2.81<br>3.04 3.19	Tyr 71<br>Trp 83<br>Tyr 337	H-pi<br>pi-pi<br>pi-pi	4.51<br>3.74<br>3.61	Tyr 73, Trp 83, Asn 84, Pro 85, Tyr 115, Gly 116, Gly 118, Thr 121, Gly 122, Leu 126, Ser 205, Trp 288, Tyr 291, Phe 297, Tyr 341, Asp 342, Trp 439, Leu 446, His 447, and Ile 451
Salvianolic acid B	-7.5902	Asp 449<br>Trp 83	H-doner<br>H-doner	2.81<br>2.70	Trp 83<br>Leu 438<br>Trp 83	pi-H<br>pi-H<br>pi-pi	3.89<br>4.09<br>3.79	Gln 68, Tyr 71, Gly 79, Glu 80, Ile 82, Trp 83, Asn 84, Pro 85, Gly 116, Gly 117, Thr 121, Gly 122, Ser 123, Leu 126, Tyr 129, Glu 204, Ser 205, Tyr 291, Phe 297, Tyr 337, Phe 338, Tyr 341, Asp 342, Thr 434, Thr 436, Ser 437, Leu 438, Trp 439, Gly 448, Asp 449, Ile 451, and Glu 452
Rosmarinic acid	-7.3476	ASN 84,<br>ASP 342	H-doner<br>H-doner	2.99<br>2.81	TYR 341	H-pi	3.81	Tyr 71, Glu 80, Trp 83, Gly 117, Gly 118, Thr 121, Glu 204, Ser 205, Tyr 291, Leu 295, Phe 297, Tyr 337, Phe 338, Leu 446, and His 447
Rosmarinic acid gluco-side	-9.4433	-	-	-	Trp 439<br>Trp 83<br>Trp 337	H-pi<br>pi-pi<br>pi-pi	4.52<br>3.65<br>3.76	Tyr 71, Gly 116, Gly 117, Gly 118, Thr 121, Leu 126, Tyr 129, Glu 204, Ser 205, Trp 288, Tyr 291, Phe 297, Phe 338, Tyr 341, Asp 342, Leu 446, His 447, Gly 448, Asp 449, and Ile 451
Salvianolic acid A	-9.2815	-	-	-	Tyr 71<br>Trp 83<br>Tyr 337	H-pi<br>pi-pi<br>pi-pi	4.02<br>3.40<br>3.94	Tyr 71, Glu 80, Asn 84, Gly 116, Gly 117, Thr 121, Tyr 129, Glu 204, Ser 205, Trp 288, Tyr 291, Phe 297, Tyr 337, Phe 338, Tyr 341, Asp 342, Leu 446, His 447, and Gly 448

A.A= Amino acid

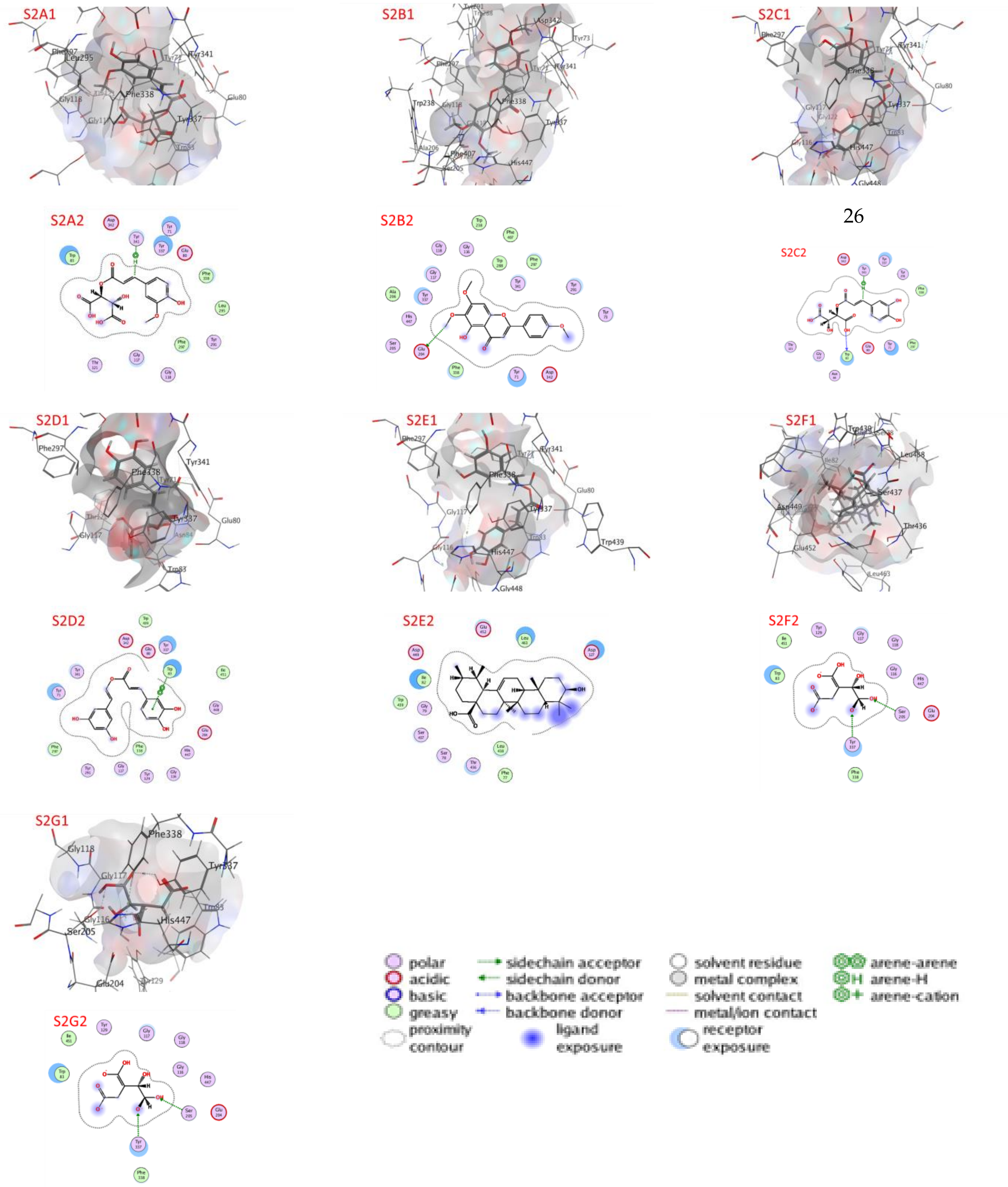

26

Figure S2. A docking image of the binding sites of Acetylcholinesterase AChE (1QON) with fertaric acid, (S2A1,2), salvigenin (S2B1,2), nepetoidin B (S2C1,2), caftaric acid (S2D1,2), nepetoidin A (S2E1,2), ursolic acid (S2F1,2), and isocitric acid (S2G1,2). Complex three-dimensional (stereoview) structures (S2A1,S2B1,S2C1,S2D1,S2E1,S2F1,S2G1,2) and two-dimensional (S2A2,S2B2,S2C2,S2D2,S2E2,S2F2,S2G2) interaction maps.

Table S3. Docking values and binding interactions of compounds with acid phosphatase (ACP) (PDB: 3IT3)

Compounds	ΔG (kcal/mol)	H-bond (donor-Acceptor)			H-pi or pi-pi bond			van der Waals
		Ligand A. A.	interaction	Distance (Å)	Ligand A.A	interaction	Distance (Å)	
Chicoric acid	-9.4951	-	-	-	TYR 130	pi-pi	3.55	Arg 11, His 12, Arg 15, Phe 18, Arg 79, Gln 127, Tye 133, Leu 134, Tye 142, Asn 173, Thr 177, Asp 180, Leu 212, Gln 215, His 255, Ala 256, and Leu 257
Quercetin-3-O-rutinoside	-8.8978	-	-	-	Phe 18<br>Tyr 130	pi-pi<br>pi-pi	3.53<br>3.65	Leu 32, Ser 33, Thr 77, Arg 79, Ala 121, Gln 127, Tyr 133, Leu 212, His 255, and Leu 257
Salvianolic acid B	-9.5805	Ala 256	H-Acceptor	3.18	Phe 18<br>Tyr 130	pi-pi<br>pi-pi	3.55<br>3.99	Arg 11, His 12, Arg 15, Ser 33, Glu 34, His 76, Thr 77, Asn 78, Arg 79, Ala 121, Gln 127, Glu 131, Ile 176, Thr 177, Asp 180, Leu 212, His 255, Ala 256, and Leu 257
Fertaric acid	-7.3173	Ala 256	H-Acceptor	3.23	Phe 18<br>Tyr 130	pi-pi<br>pi-pi	3.95<br>3.49	Arg 11, His 12, Arg 15, Arg 79, Gln 127, Tyr 133, Asp 180, Leu 212, His 255, Ala 256, and Leu 257
Rosmarinic acid gluco-side	-7.6360	-	-	-	-	-	-	Phe 18, Thr 77, Arg 79, Gln 127, Tyr 130, Leu 134, Tyr 142, Arg 168, Asn 173, Asp 174, Ile 176, and Thr 177
Salvianolic acid A	-8.3457	His 12<br>Arg 11<br>Arg 11<br>Ala 256	H-doner<br>H-acceptor<br>H-acceptor<br>H-acceptor	2.95<br>3.21<br>3.13<br>3.18	Phe 18<br>Tyr 130	pi-pi<br>pi-pi	3.69<br>3.60	Arg 15, His 76, Thr 77, Arg 79, Ser 120, Ala 121, Ser 123, Gln 127, Phe 128, Pro 129, Tye 133, Asp 180, Leu 212, His 255, and Leu 257

A.A= Amino acid

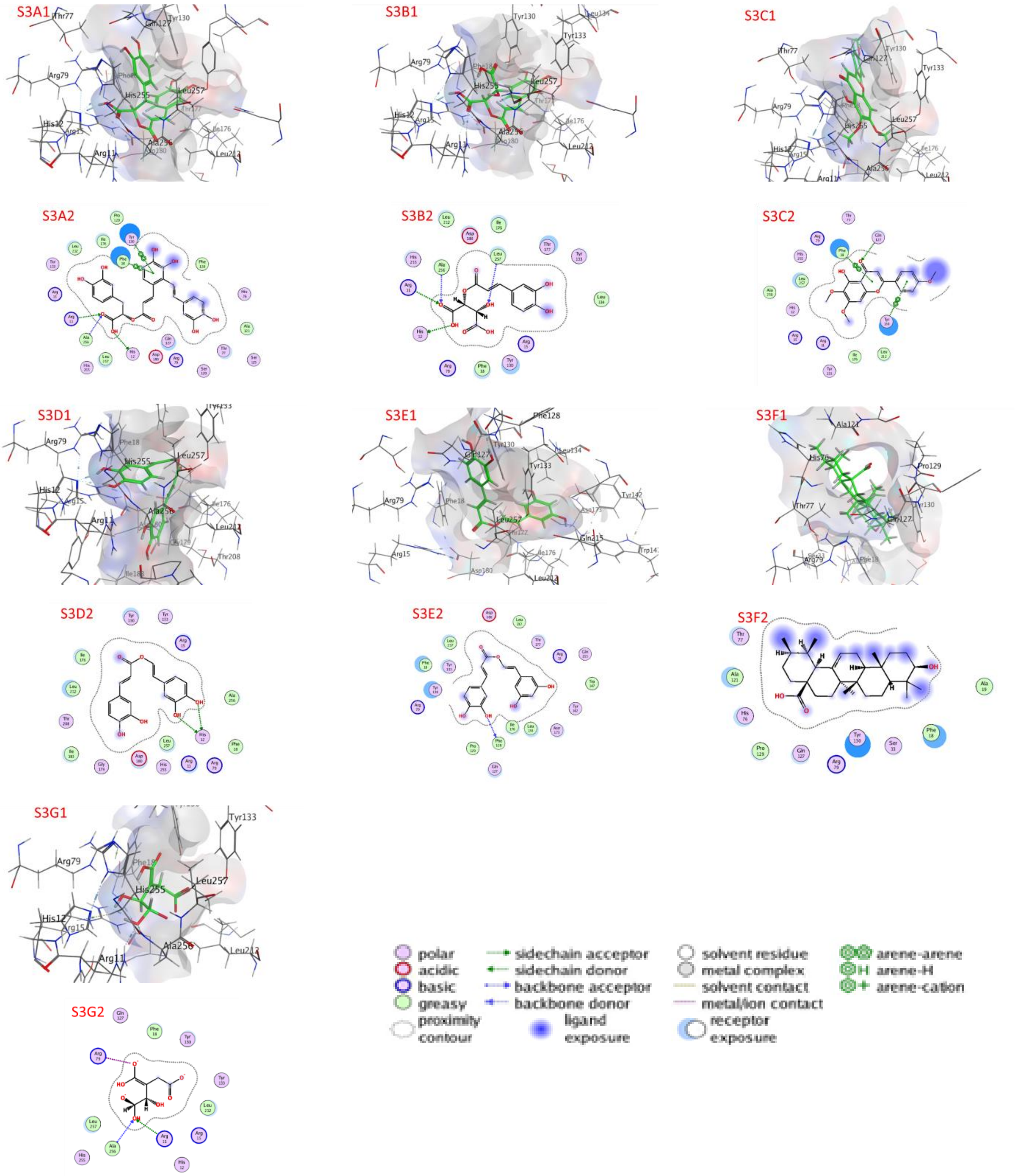

Figure S3. A docking image of the binding sites of Acid phosphatase (ACP) (3IT3) with rosmarinic acid B (S3A1,2), caftaric acid (S3B1,2), salvigenin (S3C1,2), nepetoidin B (S3D1,2), nepetoidin A (S3E1,2), ursolic acid (S3F1,2), and isocitric acid (S3G1,2). Complex three-dimensional (stereoview) structures (S3A1,S3B1,S3C1,S3D1,S3E1,S3F1,S3G1) and two-dimensional (S3A2,S3B2,S3C2,S3D2,S3E2,S3F2,S3G2) interaction maps.

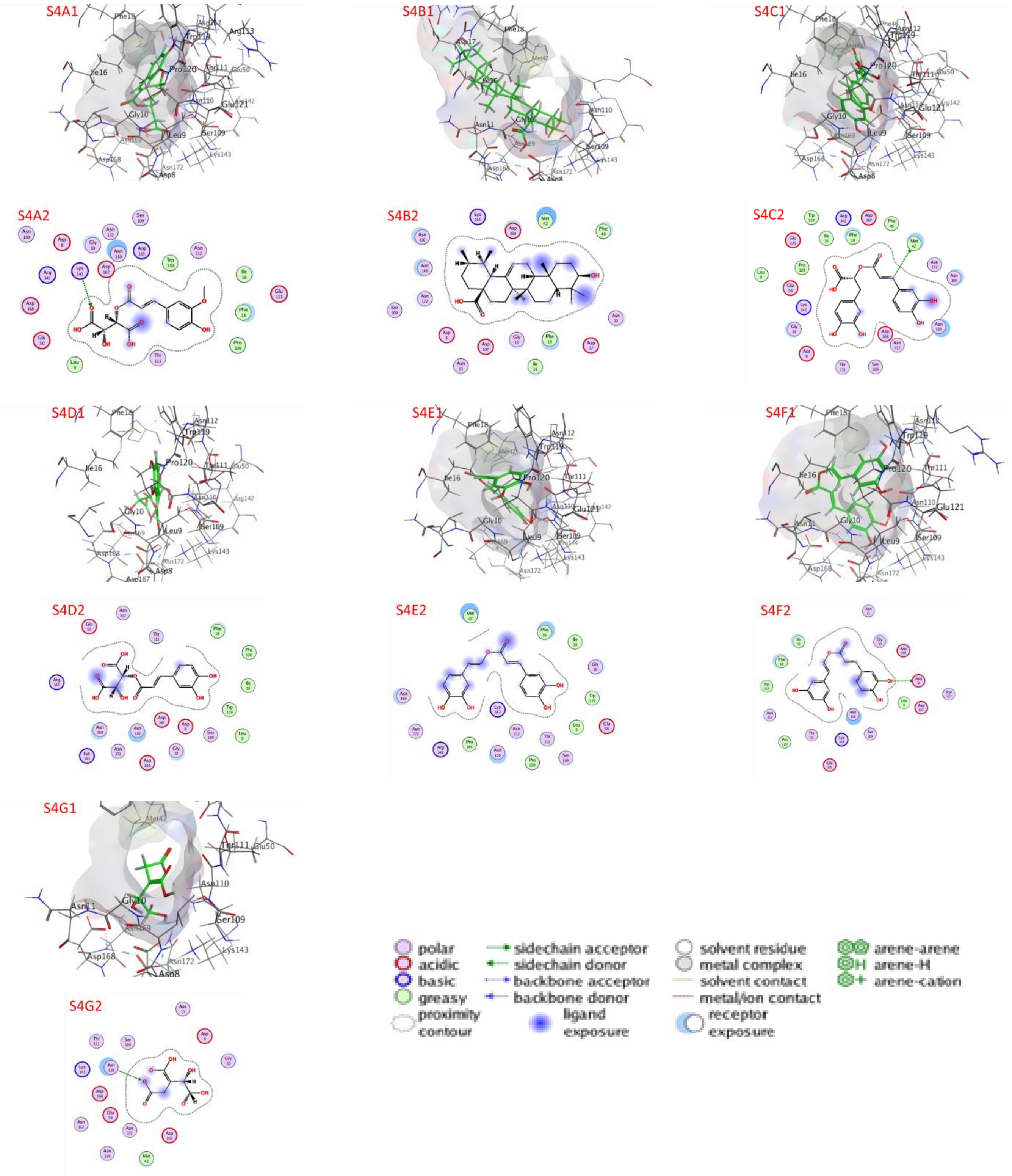

Figure S4. A docking image of the binding sites of Alkaline phosphatase (ALP) (2B0C) with fertaric acid (S4A1,2), ursolic acid (S4B1,2), rosmarinic acid (S4C1,2), caftaric acid (S4D1,2), nepetoidin B (S4E1,2), nepetoidin A (S4F1,2), and isocitric acid (GS41,2). Complex three-dimensional (stereoview) structures (S4A1,S4B1,S4C1,S4D1,S4E1,S4F1,S4G1) and two-dimensional (S4A2,S4B2,S4C2,S4D2,S4E2,S4F2.S4G2) interaction maps.

Table S4. Docking values and binding interactions of compounds with alkaline phosphatase (ALP) (PDB: 2B0C)

Compounds	ΔG (kcal/mol)	H-bond (donor-Acceptor)			H-pi or pi-pi bond			van der Waals
		Ligand A. A.	interaction	Distance (Å)	Ligand A.A	interaction	Distance (Å)	
Chicoric acid	-7.5352	Phe 18	H-acceptor	3.30	-	-	-	Asp 8, Leu 9, Gly 10, Asn 11, Ile 16, Asp 17, Asn 19, Phe 40, Met 42, Phe 46, Ser 109, Asn 110, Thr 111, Asn 112, Glu 121, Lys 143, Asp 167, Asp 168, Asn 169, and Asn 172
Quercetin-3-O-rutinoside	-8.9556	Asn 110<br>Asp 167<br>Asn 110<br>Lys 143	H-doner<br>H-doner<br>H-acceptor<br>H-acceptor	3.26<br>3.07<br>2.99<br>3.38	-	-	-	Asp 8, Gly 10, Asn 11, Ile 16, Phe 18, Phe 40, Met 42, Ser 109, Thr 111, Asn 112, Trp 119, Arg 142, Pro 144, Asp 168, Asn 169, Asp 171, and Asn 172
Salvianolic acid B	-8.8400	Met 42<br>Asp 8<br>Asp 168	H-doner<br>H-doner<br>H-doner	3.74<br>3.10<br>3.09	Phe 18	pi-pi	3.69	Gly 10, Asn 11, Ile 16, Asp 17, Asn 19, Phe 40, Phe 46, His 47, Glu 50, Ser 109, Asn 110, The 111, Asn 112, Trp 119, Arg 142, Asp 167, Asn 169, and Asn 172
Salvigenin	-7.6869	-	-	-	Thr 111	pi-H	4.31	Asp 8, Leu 9, Gly 10, Thr 11, Asn 12, Ile 16, Phe 18, Met 42, Phe 46, His 47, Glu 50, Ser 109, Asn 110, Asn 112, Trp 119, Pro 120, Arg 142, Lys 143, Pro 144, Asp 167, Asp 168, Asn 169, and Asn 172
Rosmarinic acid glucoside	-7.7995	Asn 110<br>Asp 168	H-doner<br>H-doner	3.01<br>3.08	-	-	-	Asp 8, Leu 9, Gly 10, Asn 11, Ile 16, Phe 18, Asn 19, Lys 37, Phe 40, Met 42, Ser 109, Thr 111, Asn 112, Trp 119, Lys 143, Asp 167, Asn 169, and Asn 172
Salvianolic acid A	-8.0411	-	-	-	-	-	-	Leu 9, Gly 10, Ile 16, Asp 17, Phe 18, Met 42, Glu 50, Ser 109, Asn 110, The 111, Asn 112, Trp 119, Glu 121, Arg 142, Lys 143, Pro 144, Asp 167, Asp 168, Asn 169, and Asn 172.

A.A= Amino acid

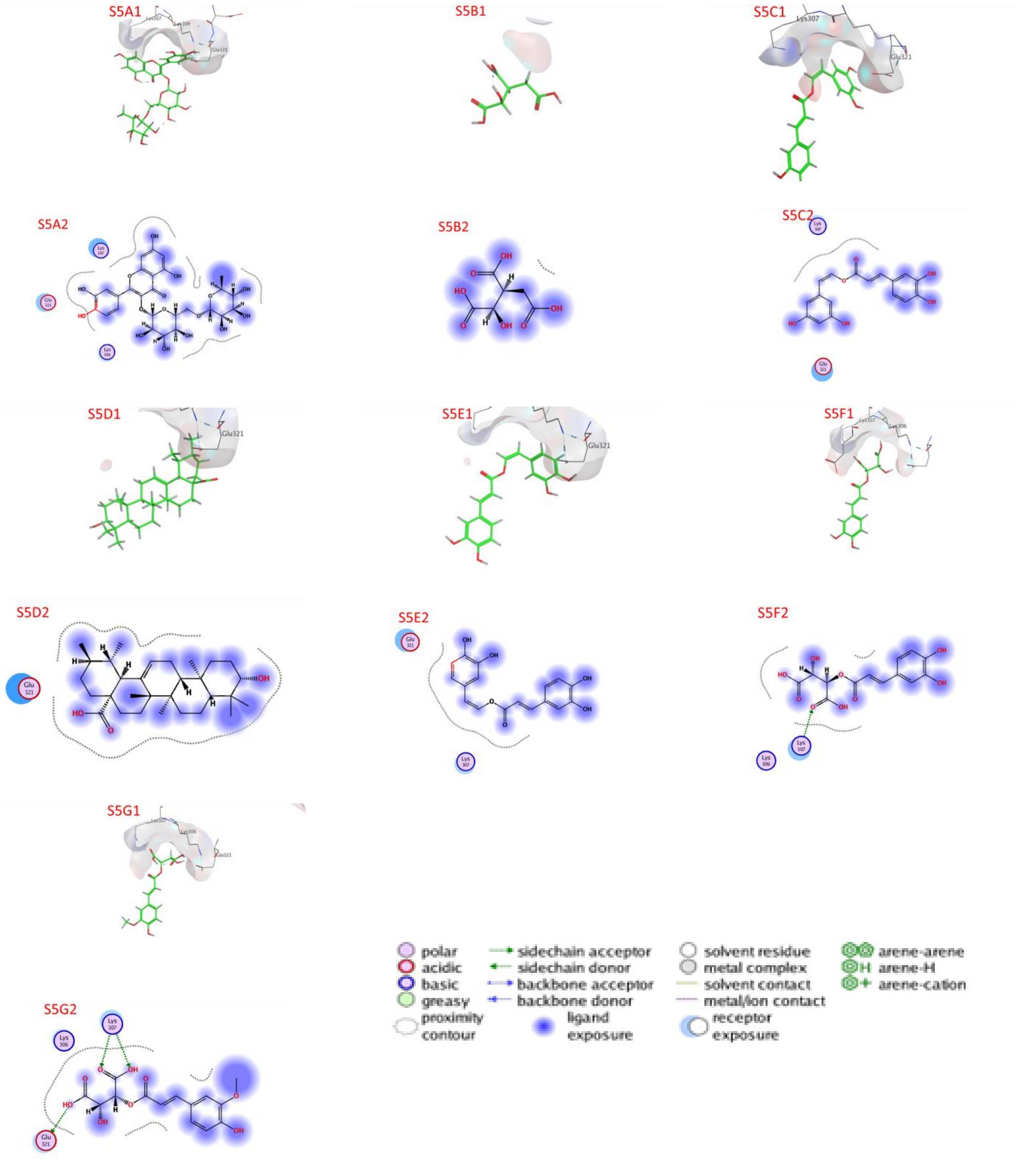

Figure S5. A docking image of the binding sites of Gamma-aminobutyric acid-transaminase (GABA-T) (3IP9) with quercetin-3-O-rutinoside (S5A1,2), isocitric acid (S5B1,2), nepetoidin A (S5C1,2), ursolic acid (S5D1,2), nepetoidin B (S5E1,2), caftaric acid (S5F1,2), and fetaric acid (S5G1,2). Complex three-dimensional (stereoview) structures (S5A1,S5B1,S5C1,S5D1,S5E1,S5F1,S5G1) and two-dimensional (S5A2,S5B2,S5C2,S5D2,S5E2,S5F2,S5G2) interaction maps

Table S5. Docking values and binding interactions of compounds with alkaline phosphatase (GABA-T) (PDB: 3IP9)

Compounds	ΔG (kcal/mol)	H-bond (donor-Acceptor)			H-pi or pi-pi bond			van der Waals
		Ligand A. A.	interaction	Distance (Å)	Ligand A.A	interaction	Distance (Å)	
Chicoric acid	-4.8675	Ala 302	H-doner	2.87	-	-	-	Glu 303, Leu 305, Lys 306, Lys 307, Phe 115, Phe 319, Asp 320, and Glu 321
rosmarinic acid	-4.9175	Lys 307	H-acceptor	3.31	-	-	-	Glu 303, Lys 306, and Glu 321
Salvianolic acid B	-5.7478	Lys 307	H-acceptor	3.65	-	-	-	Lys 306 and Glu 312
Salvigenin	-7.1403	-	-	-	-	-	-	Lys 306 and Glu 312
Rosmarinic acid glucoside	-	-	-	-	-	-	-	Glu 321
Salvianolic acid A	-5.3285	-	-	-	-	-	-	Glu 303, Lys 306, and Glu 312

A.A= Amino acid

Table S6. Experimental chemicals' ADMET evaluation.

Compounds	HBD	HBA	LogP	LogS (µg/mL)	BBB	PPB	CYP450 2D6 substrate	H-HT	TPSA
rosmarinic acid	5	7	1.761	96.535	++	+	+	+	144.52
Nepetoidin A	4	5	2.736	47.026	-	+	-	+	107.22
Nepetoidin B	4	6	2.736	47.026	-	+	-	+	107.22
Ursolic acid	2	2	7.09	0.187	++	+	-	---	57.53
Salvigenin	1	6	3.191	44.905	+	+	++	++	78.13
Quercetin-3-O-rutinoside	10	16	-1.687	315.283	---	+	---	---	269.43
Rosmarinic acid glucoside	8	13	-0.98	473.208	---	+	+	-	223.67
chicoric acid	6	10	1.228	97.526	+	+	-	-	208.12
Isocitric acid	4	7	-1.393	42518	+++	+	-	-	132.13
Caftaric acid	5	7	-0.447	1847.003	-	+	-	+	161.59
Fertaric acid	4	7	-0.144	2520.924	-	+	+	++	150.59
Salvianolic acid A	7	9	3.343	11.916	+	+	-	-	184.98
Salvianolic acid B	9	14	3.335	34.08	++	+	+	---	278.04

HBD, HBA: hydrogen bond donor and acceptor; LogS: Solubility; LogP: Distribution Coefficient P; PPB: Plasma Protein Binding; BBB: Blood–Brain Barrier; H-HT: Human Hepatotoxicity. LogS: optimal, low, moderate, and high solubility with value > -4, <10, 10–60, >60 µg/mL; LogP: optimal, poor lipid bilayer permeability, poor aqueous solubility with value 0-3, <0, and >3; BBB+ or – with >=0.1, <0.1; classification of models & probability 0~0.1 (---), 0.1~0.3 (--), 0.3~0.5 (-), 0.5~0.7 (+), 0.7~0.9 (++), 0.9~1 (+++).
